# Organocatalytic asymmetric selenofunctionalization of tryptamine for the synthesis of hexahydropyrrolo[2,3-*b*]indole derivatives

**DOI:** 10.3762/bjoc.9.177

**Published:** 2013-08-01

**Authors:** Qiang Wei, Ya-Yi Wang, Yu-Liu Du, Liu-Zhu Gong

**Affiliations:** 1 Hefei National Laboratory for Physical Sciences at the Microscale and Department of Chemistry, University of Science and Technology of China, Hefei, 230026, China

**Keywords:** catalysis, chiral phosphoric acid, hexahydropyrrolo[2,3-*b*]indole, indole alkaloids, natural products, selenofunctionalization

## Abstract

A chiral phosphoric acid-catalyzed selenofunctionalization of tryptamine derivatives provides access to 3a-(phenylselenyl)-1,2,3,3a,8,8a-hexahydropyrrolo[2,3-*b*]indole derivatives in high yields and with synthetically useful levels of enantioselectivity (up to 89% ee).

## Introduction

Selenofunctionalization of carbon–carbon double bonds provides practicable opportunities for rapid construction of molecule complexity [1–8], because the versatile carbon–selenium bond could either stabilize carbanions [9–10], serve as a radical precursor [11–13], or undergo a *syn*-selective oxidative elimination via the selenoxide [14–15]. A widely accepted mechanism suggests that a key discrete seleniranium ion intermediate is initially formed, and then trapped by internal amine through nucleophilic attack to furnish the product. So far, in addition to the chiral-substrate-induced strategy [16], chiral selenylating agents [17–24] are commonly designed for asymmetric selenofunctionalization of carbon–carbon double bonds. In 2010, Denmark and co-workers reported a Lewis base catalyzed asymmetric selenoetherification of olefins, whereas the enantioselectivity was not quite synthetically attractive (up to 70% ee) [25]. As chiral 3-substituted hexahydropyrroloindoline is a key structural moiety prevalent in a large number of bioactive indole alkaloids [26–27], direct access to which by selenofunctionalization has been considered to be promising but challenging. Danishefsky found that the treatment of bis(Cbz)tryptamine with *N*-phenylselenophthalimide (*N*-PSP) in the presence of a catalytic amount of p-toluenesulfonic acid (PTSA) was able to afford a racemic selenofunctionalization product in 84% yield [28]. These leading findings indicate that either Lewis base or Brønsted acid shows catalytic activity for the selenofunctionalization reaction. Since chiral phosphoric acids have been shown to be Brønsted acid/Lewis base bifunctional organocatalysts [29–33], we ask whether the chiral BINOL-based phosphoric acids are able to catalyze the selenofunctionalization of tryptamine derivatives.

## Results and Discussion

**Figure 1:**
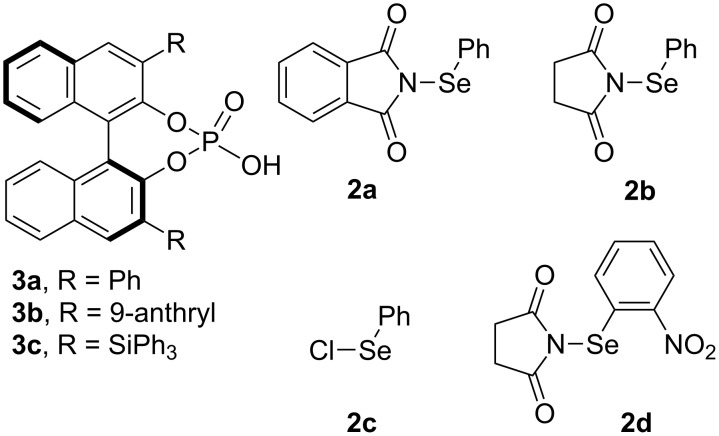
Catalysts and seleno reagents evaluated in this study.

Initially, we investigated a reaction of bis(Cbz)tryptamine reagent **1a** with *N*-phenylselenophthalimide (*N*-PSP) (**2a**) by using phosphoric acids **3** (Table 1, Figure 1) as catalysts for the validation of our hypothesis. Encouragingly, the reaction proceeded smoothly in the presence of 10 mol % of the phosphoric acids evaluated under the assistance of 5 Å molecular sieves. Apparently, the stereoselectivity depended on the N-protecting group of tryptamine **1**. When nitrogen atoms of the tryptamine were both protected with Cbz, a very poor enantioselectivity was observed regardless of the catalysts used (Table 1, entries 1–3). Notably, only the substrate bearing an electron-withdrawing N-protecting group at the indole nitrogen (R^2^) underwent a smooth reaction to afford the desired product. When the R^2^ was replaced with a methyl group, the *N*-PSP directly underwent a coupling reaction with tryptamine derivatives **1** at the 2-position in 76% yield [34], indicating that the electronically rich substitution inhibited the desired selenofunctionalization reaction. Other selenofunctionalization reagents, such as **2b** and **2c** could also participate in the reaction under similar conditions, but both showed lower reactivity than **2a**. Interestingly, the selenofunctionalization reagent **2d**, which was the best substrate in the reaction developed by Denmark [25], however, was completely unreactive in our case. After the optimal R^2^ protecting group and the phenylseleno reagent were determined, we focused on the evaluation of the N-protecting group of the tryptamine (R^1^) to improve the enantioselectivity. The Fmoc group was found to be better than any other substituents that were screened (Table 1, entries 10 and 11 versus 12). The optimization of reaction parameters including solvents and temperature found dichloroethane (DCE) to be the best solvent in terms of enantioselectivity, and the best results could be accessed by conducting at 0 °C (Table 1, entries 13 and 14).

**Table 1:** Optimization of reaction conditions.^a^

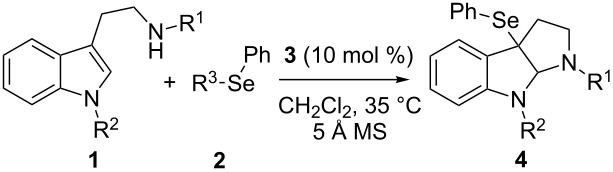						

entry	**3**	R^1^	R^2^	**2**	yield (%)^b^	ee (%)^c^

1	**3a**	Cbz	Cbz	**2a**	56	10
2	**3b**	Cbz	Cbz	**2a**	46	6
3	**3c**	Cbz	Cbz	**2a**	36	0
4	**3a**	Cbz	Boc	**2a**	49	6
5	**3a**	Cbz	Me	**2a**	–	–
6	**3a**	Cbz	Ac	**2a**	67	24
7	**3a**	Cbz	Ac	**2b**	63	25
8	**3a**	Cbz	Ac	**2c**	37	5
9	**3b**	Cbz	Ac	**2d**	–	–
10	**3b**	Cbz	Ac	**2a**	65	48
11	**3b**	CO_2_Et	Ac	**2a**	61	27
12	**3b**	Fmoc	Ac	**2a**	60	63
13^d^	**3b**	Fmoc	Ac	**2a**	75	77
14^d,e^	**3b**	Fmoc	Ac	**2a**	78	86

^a^The reaction was performed in 0.1 mmol scale in DCM (1 mL) with 5 Å MS (100 mg). ^b^Isolated yield. ^c^The ee was determined by HPLC. ^d^In DCE. ^e^The temperature was 0 °C.

**Figure 2:**
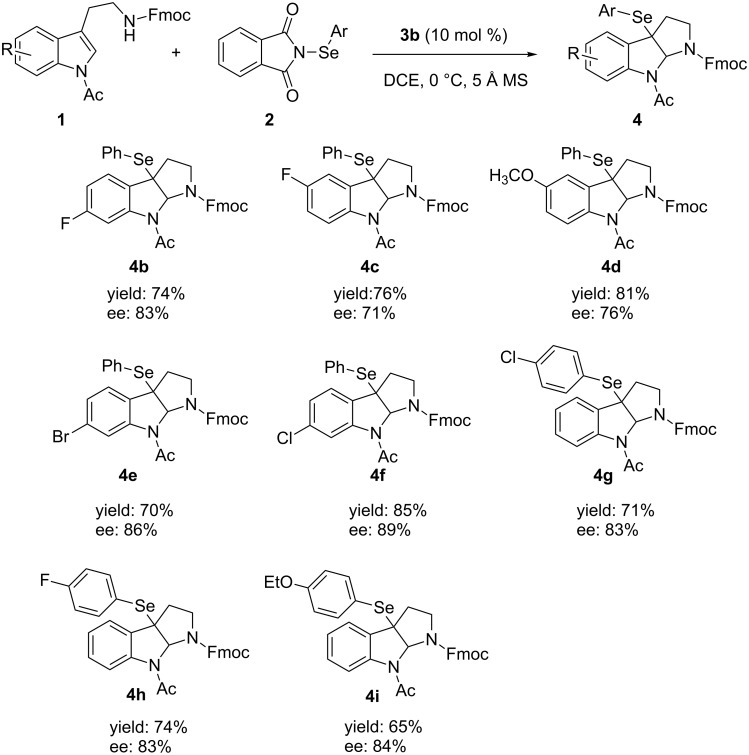
Generality for substitution at the indoline moiety. The reaction was performed in 0.1 mmol scale in DCE (1 mL) with 5 Å MS (100 mg) in 0 °C and the ratio of **2**/**1** is 3:1. The reaction was performed for 1–3 days. The given yields are isolated yields, and the ee’s were determined by HPLC.

**Figure 3:**
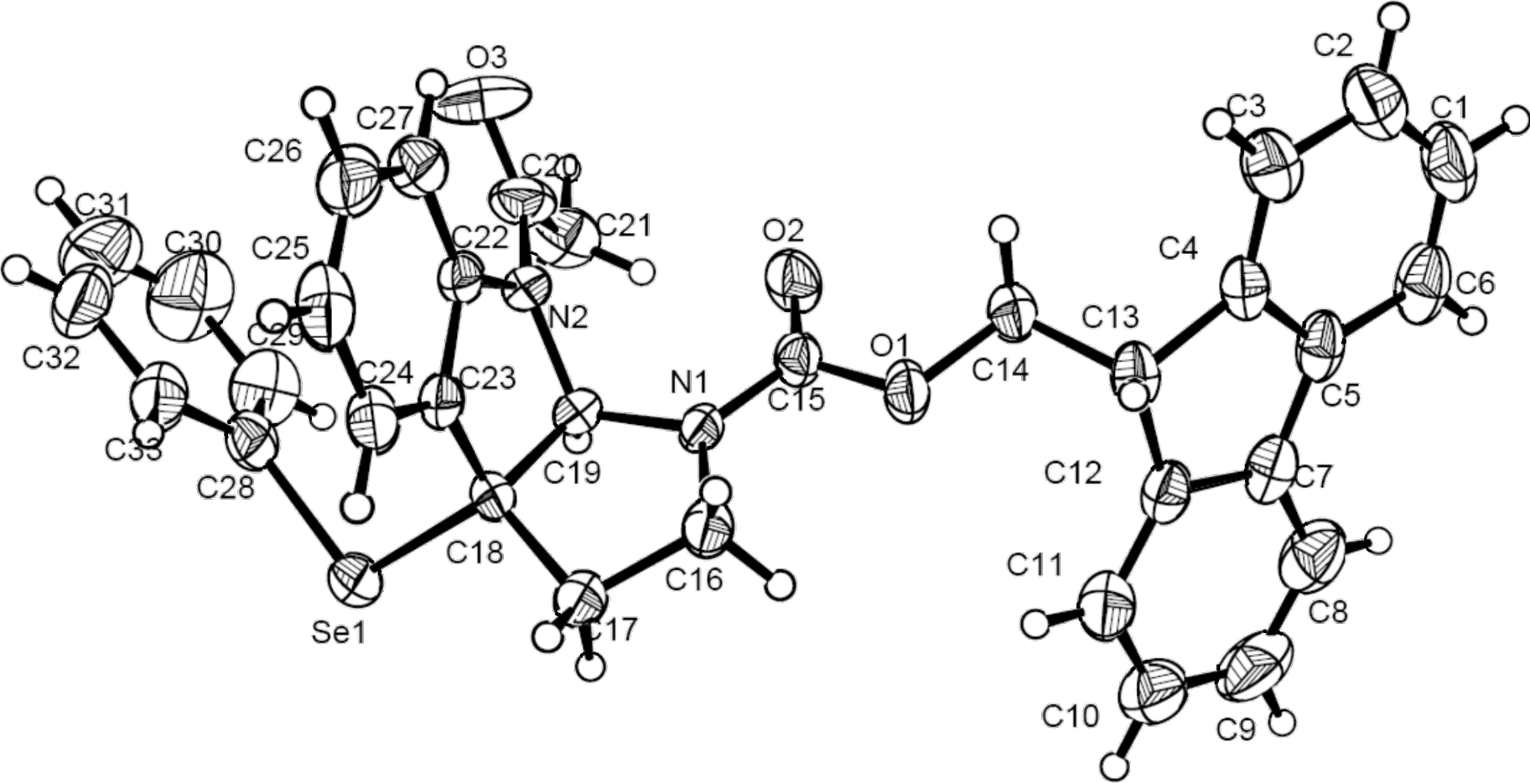
X-ray crystallography of **4a** catalyzed by (*S*)-**3b**.

After optimizing the reaction conditions, a variety of tryptamine analogues were synthesized for this chiral phosphoric acid-catalyzed asymmetric selenofunctionalization. As shown in Figure 2, no matter what the chemical and electronic feature of the substituents on the benzene moiety of either substrates or *N*-PSP, various tryptamine analogues could be smoothly transformed into the corresponding products in satisfactory yields (65–85%) and with good enantioselectivities (71–89%; Figure 2, **4b**–**4i**). In addition, the products were solid and easy to recrystallize to enhance the optical purity. After a single recrystallization from methanol, the optical purity of some products, such as **4e**, was enhanced to >99% ee. Importantly, the configuration could be assigned by X-ray crystallography. The crystal structure of **4a** (>99%) indicated that the configuration of the stereogenic centers was assigned to be (3a*R*,8a*S*) (Figure 3) [35].

**Scheme 1:**
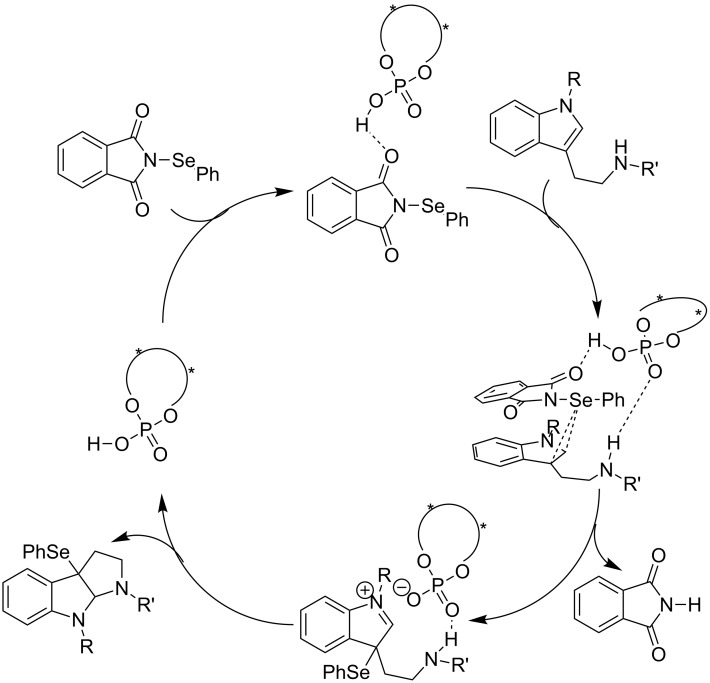
The plausible reaction mechanism.

On the basis of experimental observations, we proposed a reaction mechanism (Scheme 1). The phosphoric acid acts as a bifunctional catalyst and simultaneously activates both the tryptamine derivative and *N*-PSP by hydrogen-bonding interaction. Then, the asymmetric selenofunctionalization occurred at the 3-substituted tryptamine and subsequently the proton of the phosphoric acid protonates the phthalimide anion to release phthalimide. Finally, the amide on the side chain of the tryptamine derivatives attacks the resultant iminium cation leading to the formation of the product in an enantioselective manner.

**Scheme 2:**
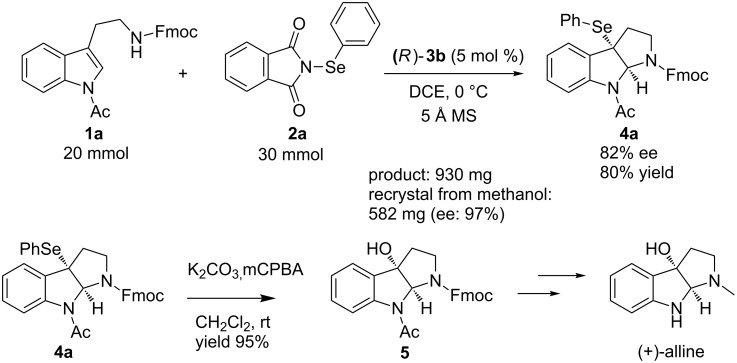
Scale up of the protocol and synthetic application.

Finally, we demonstrate the synthetic application of this reaction in the construction of the 3a-(phenylselenyl)bispyrrolidino[2,3-*b*]indoline core structure (Scheme 2). Under the optimized reaction conditions, the enantioselective substitution reaction gave **4a** in 78% yield and 86% ee. However, when the reaction was scaled up to 20 mmol, both the yield and the stereoselectivity were significantly sacrificed. To our delight, the reaction running on a similar scale could give **4a** in 80% yield and 82% ee by tuning the stoichiometry of the phenylseleno reagent (**2a**) to 1.5 equiv by using 5 mol % of catalyst (*R*)-**3b**. After a single recrystallization from methanol, the product **4a** was obtained in 50% yield and with 97% ee. The oxidative deselenation of **4a** with MCPBA afforded the corresponding alcohol **5** in 95% yield. The stereochemistry of the alcohol was found to be identical to that of the parent selenide, as demonstrated previously [36].

## Conclusion

In summary, we have developed a reaction for the enantioselective selenofunctionalization of tryptamine derivatives with *N*-phenylselenophthalimide (*N*-PSP) catalyzed by chiral phosphoric acids (up to 89% ee). In this context, we used this protocol to prepare the key chiral precursor of the (+)-alline.

## Supporting Information

File 1 Experimental procedures and characterization data for new compounds. In addition, confirmatory crystallographic data are included.
