# Supplementary material for: Organocatalytic asymmetric selenofunctionalization of tryptamine for the synthesis of hexahydropyrrolo[2,3-b]indole derivatives
Source: Beilstein J Org Chem. 2013 Aug 1;9:1559–64. doi: 10.3762/bjoc.9.177 (PMC3740605; doi:10.3762/bjoc.9.177)
Supplement: File 1 — Experimental procedures and characterization data for new compounds. In addition, confirmatory crystallographic data are included. [file Beilstein_J_Org_Chem-09-1559-s001.pdf]

Supporting Information

for

Organocatalytic asymmetric selenofunctionalization of tryptamine for the synthesis of hexahydropyrrolo[2,3-*b*]indole derivatives

Qiang Wei, Ya-Yi Wang, Yu-Liu Du and Liu-Zhu Gong*

Address: Hefei National Laboratory for Physical Sciences at the Microscale and Department of Chemistry, University of Science and Technology of China, Hefei, 230026, China

Email: Liu-Zhu Gong - gonglz@ustc.edu.cn

Experimental part

General data: NMR spectra were recorded on a Brucker 400 MHz spectrometer. Mass spectra were recorded on a Thermo LTQ Orbitrap XL (ESI^+). Infrared spectra were recorded on a Nicolet MX-1E FTIR spectromter. HPLC analysis was performed on Waters-Breeze (2487 Dual λ Absorbance Detector and 1525 Binary HPLC Pump, UV detection monitored at 205 nm or 254 nm). Chiralpak OD and IA, columns were purchased from Daicel Chemical Industries, LTD. Dichloromethane and 1,2-dichloroethane were dried over CaH_2 and distilled prior to use. Hexane and ethyl acetate for the column chromatography were distilled before use. The relative and absolute configurations of **4a** were assigned by the X-ray analysis.

Materials: All starting materials were purchased from Acros and Aldrich and used directly.

The tryptamine was prepared according to literature methods, purified by chromatography and recrystallization from ethanol.

General procedure for tryptamine derivatives

A mixture of tryptamine (5 mmol) and Et_3N (6 mmol) in CH_2Cl_2 (40 mL) was stirred at 0 °C, and then FmocCl (7 mmol) was added with a spoon. The reaction mixture was stirred at room temperature until the reaction was complete (the reaction time was 1–4 hours). The resultant solution was washed with water and extracted with EtOAc. The combined organic layers were washed with brine and dried over anhydrous Na_2SO_4. The crude product was purified through flash column chromatography on silica gel (eluent: petroleum ether:ethyl acetate = 4:1–2:1) to yield pure products (Fmoc-2-(1*H*-indol-3-yl)ethylcarbamate), yield 84%.

To a mixture of Fmoc-2-(1*H*-indol-3-yl)ethylcarbamate (4 mmol) and Bu_4NHSO_4 (0.8 mmol) in CH_2Cl_2 (30 mL) were sequentially added Ac_2O (8 mmol) and aqueous NaOH solution (2 mol/L, 5 mL) at 0 °C via sryinge. The reaction mixture was stirred at room temperature until the reaction was complete (the reaction time was 5–15 minutes; the Fmoc group would be removed for a long time). The resultant solution was poured into a mixture of water and EtOAc (1/1 ratio). The aqueous layer was extracted with EtOAc (3 × 40 mL). The combined organic layers were washed with brine and dried over anhydrous Na_2SO_4. The crude product was purified through flash column chromatography on silica gel (eluent: petroleum ether:ethyl acetate = 3:1–2:1) to give products (Fmoc-2-(1-acetyl-1*H*-indol-3-yl) ethylcarbamate), yield: 91%.

(9*H*-Fluoren-9-yl)methyl 2-(1-acetyl-1*H*-indol-3-yl)ethylcarbamate (1a).

^{1}H NMR ($CDCl_3$, 400 MHz) δ (ppm): 2.57 (s, 3H), 2.93 (m, 2H), 3.56 (m, 2H), 4.20 (t, *J* = 6.72 Hz, 1H), 4.43 (d, *J* = 6.72 Hz, 2H), 4.88 (m, 1H), 7.28 (m, 4H), 7.39 (m, 3H), 7.55 (m, 3H), 7.75 (d, *J* = 7.52 Hz, 2H), 8.43 (d, *J* = 8.04 Hz, 1H).

(9*H*-Fluoren-9-yl)methyl 2-(1-acetyl-6-fluoro-1*H*-indol-3-yl)ethylcarbamate (1b).

^{1}H NMR ($CDCl_3$, 400 MHz) δ (ppm): 2.55 (s, 3H), 2.89 (m, 2H), 3.55 (m, 2H), 4.21 (t, *J* = 6.70 Hz, 1H), 4.43 (d, *J* = 6.70 Hz, 2H), 4.93 (m, 1H), 7.27 (m, 3H), 7.38 (m, 3H), 7.55 (d, *J* = 7.44 Hz, 2H), 7.75 (m, 3H), 8.58 (s, 1H).

(9*H*-Fluoren-9-yl)methyl 2-(1-acetyl-5-fluoro-1*H*-indol-3-yl)ethylcarbamate (1c).

^{1}H-NMR ($CDCl_3$, 400 MHz) δ (ppm): 2.54 (s, 3H), 2.89 (m, 2H), 3.54 (m, 2H), 4.21 (t, *J* = 6.72 Hz, 1H), 4.44 (d, *J* = 6.72 Hz, 2H), 4.87 (m, 1H), 7.28 (m, 3H), 7.39 (m, 3H), 7.52 (d, *J* = 7.40 Hz, 2H),

7.63 (s, 1H), 7.75 (d, *J* = 7.52 Hz, 2H), 8.30 (d, *J* = 8.8 Hz, 1H).

(9*H*-Fluoren-9-yl)methyl 2-(1-acetyl-5-methoxy-1*H*-indol-3-yl)ethylcarbamate (1d).
^{1}H NMR ($CDCl_3$, 400 MHz) δ (ppm): 2.53 (s, 3H), 2.89 (m, 2H), 3.55 (m, 2H), 3.84 (s, 3H), 4.21 (t, *J* = 6.76 Hz, 1H), 4.42 (d, *J* = 6.76 Hz, 2H), 4.93 (m, 1H), 6.95 (m, 2H), 7.21 (s, 1H), 7.27 (t, *J* = 7.04, Hz, 2H), 7.38 (t, *J* = 7.44 Hz, 2H), 7.55 (d, *J* = 7.44 Hz, 2H), 7.75 (d, *J* = 7.04 Hz, 2H), 8.32 (d, *J* = 7.12, 1H).

(9*H*-Fluoren-9-yl)methyl 2-(1-acetyl-6-bromo-1*H*-indol-3-yl)ethylcarbamate (1e).
^{1}H NMR ($CDCl_3$, 400 MHz) δ (ppm): 2.54 (s, 3H), 2.90 (m, 2H), 3.52 (m, 2H), 4.20 (t, *J* = 6.72 Hz, 1H), 4.43 (d, *J* = 6.72 Hz, 2H), 4.87 (m, 1H), 7.21 (s, 1H), 7.28 (t, *J* = 7.48 Hz, 2H), 7.39 (m, 4H), 7.55 (d, *J* = 7.44 Hz, 2H), 7.75 (d, *J* = 7.48 Hz, 2H), 8.64 (s, 1H).

(9*H*-Fluoren-9-yl)methyl 2-(1-acetyl-6-chloro-1*H*-indol-3-yl)ethylcarbamate (1f).
^{1}H NMR ($CDCl_3$, 400 MHz) δ (ppm): 2.54 (s, 3H), 2.89 (m, 2H), 3.54 (m, 2H), 4.20 (t, *J* = 6.64 Hz, 1H), 4.43 (d, *J* = 6.64 Hz, 2H), 4.93 (m, 1H), 7.27 (m, 3H), 7.38 (m, 3H), 7.55 (d, *J* = 7.40 Hz, 2H), 7.75 (m, 3H), 8.64 (s, 1H).

General procedure for direct catalytic, asymmetric synthesis of (9*H*-fluoren-9-yl)methyl 8-acetyl-3a-(phenylselenyl)-3,3a,8,8a-tetrahydropyrrolo[2,3-*b*]indole-1(2*H*)-carboxylate
A mixture of an *N*-phenylselenophthalimide (*N*-PSP) (0.3 mmol), catalyst **3b** (0.01 mmol, 7 mg), Fmoc-Ac-tryptamine (0.1 mmol), and 5 Å molecular sieves (100 mg) was prepared in a Schlenk tube. The tube was flushed by Argon gas three times. Then, DCE (1.0 mL) was added, and the mixture was stirred at 0 °C until the reaction was complete (the reaction time was 1–3 d, monitored by TLC). The resultant solution was purified through flash column chromatography on silica gel (eluent: petroleum ether:ethyl acetate = 8:1–4:1) to yield pure products.

(9*H*-Fluoren-9-yl)methyl 8-acetyl-3a-(phenylselenyl)-3,3a,8,8a-tetrahydropyrrolo[2,3-*b*]indole-1(2*H*)-carboxylate (4a)
47 mg (Flash column chromatography eluent, petroleum ether/ethyl acetate = 6/1–4/1), 71% yield,

^{1}H NMR ($CDCl_3$, 400 MHz) δ (ppm): 2.28 (m, 4H), 2.50 (m, 1H), 2.89 (m, 1H), 3.65 (m, 1H), 4.20 (m, 1H), 4.44 (m, 2H), 6.05 (s, 1H), 7.16 (m, 4H), 7.30 (m, 4H), 7.39 (m, 2H), 7.50 (m, 2H), 7.75 (m, 2H), 7.86 (m, 2H);

^{13}C NMR (D-DMSO, 100 MHz) δ (ppm): 22.8, 34.6, 45.8, 46.5, 55.7, 66.7, 83.8, 115.9, 119.4, 120.0, 124.9, 125.3, 126.7, 127.0, 127.6, 129.1, 129.6, 131.5, 135.3, 136.7, 140.7, 140.8, 141.0, 143.4, 143.6, 153.6, 169.4;

Enantiomeric excess: 86%, determined by HPLC (Daicel Chirapak IA-H, hexane/isopropanol = 70/30, flow rate 1.0 mL/min, T = 30 °C, 254 nm): t_R = 11.93 min(minor), t_R = 25.89 min(major); IR (KBr): 2921, 2858, 1740, 1709, 1652, 1507, 1362, 1248, 1166, 831, 755, 699; HRMS exact mass calcd for $(C_{33}H_{28}N_2O_3Se + H)^+$ requires m/z 581.1343, found 581.1337.

(9*H*-Fluoren-9-yl)methyl 8-acetyl-6-fluoro-3a-(phenylselenyl)-3,3a,8,8a-tetrahydropyrrolo[2,3-*b*]indole-1(2*H*)-carboxylate (4b)

52 mg (Flash column chromatography eluent, petroleum ether/ethyl acetate = 6/1–4/1), 85% yield,

^{1}H NMR (D-DMSO, 400 MHz) δ (ppm): 2.28 (m, 4H), 2.67 (m, 2H), 3.52 (m, 1H), 4.35 (m, 3H), 5.97 (s, 1H), 7.40 (m, 11H), 7.58 (m, 3H), 7.88 (m, 2H);

^{13}C NMR (D-DMSO, 100 MHz) δ (ppm): 22.8, 34.7, 45.7, 46.6, 66.6, 84.2, 117.3 120.0, 124.3, 124.9, 125.3, 125.4, 127.0, 127.6, 129.1, 129.6, 131.8, 132.5, 132.8, 136.6, 140.7, 142.8, 143.4, 143.6, 153.6, 169.6;

Enantiomeric excess: 83%, determined by HPLC (Daicel Chirapak IA-H, hexane/isopropanol = 70/30, flow rate 1.0 mL/min, T = 30 °C, 254 nm): t_R = 9.14 min(minor), t_R = 17.04 min(major); IR (KBr): 2921, 2858, 1740, 1709, 1652, 1507, 1362, 1248, 1166, 831, 755, 699; HRMS exact mass calcd for $(C_{33}H_{27}FN_2O_3Se + H)^+$ requires m/z 599.1244, found 599.1253.

(9*H*-Fluoren-9-yl)methyl 8-acetyl-5-fluoro-3a-(phenylselenyl)-3,3a,8,8a-tetrahydropyrrolo[2,3-*b*]indole-1(2*H*)-carboxylate (4c)

46 mg (Flash column chromatography eluent, petroleum ether/ethyl acetate = 6/1–4/1), 76% yield,

^{1}H NMR (D-DMSO, 400 MHz) δ (ppm): 2.28 (m, 4H), 2.73 (m, 2H), 3.54 (m, 1H), 4.30 (m, 3H), 5.97 (s, 1H), 7.35 (m, 10H), 7.58 (m, 3H), 7.88 (m, 3H);

^{13}C NMR (D-DMSO, 100 MHz) δ (ppm): 22.8, 34.6, 45.8, 46.5, 55.8, 66.7, 83.9, 119.0, 120.0, 122.9, 123.8, 124.9, 125.3, 127.0, 127.6, 128.1, 128.7, 129.1, 129.6, 132.6, 134.2, 136.7, 140.7,

143.4, 143.6, 153.6, 169.1;

Enantiomeric excess: 71%, determined by HPLC (Daicel Chirapak IA-H, hexane/isopropanol = 70/30, flow rate 1.0 mL/min, T = 30 °C, 254 nm): t_R = 11.49 min(minor), t_R = 24.01 min(major); IR (KBr): 2921, 2858, 1740, 1709, 1652, 1507, 1362, 1248, 1166, 831, 755, 699; HRMS exact mass calcd for $(C_{33}H_{27}FN_2O_3Se + H)^+$ requires m/z 599.1244, found 599.1250.

(9*H*-Fluoren-9-yl)methyl 8-acetyl-5-methoxy-3a-(phenylselenyl)-3,3a,8,8a-tetrahydropyrrolo[2,3-*b*]indole-1(2*H*)-carboxylate (4d)

49 mg (Flash column chromatography eluent, petroleum ether/ethyl acetate = 6/1–4/1), 81% yield,

^{1}H NMR ($CDCl_3$, 400 MHz) δ (ppm): 2.27 (m, 4H), 2.71 (m, 2H), 3.51 (m, 1H), 3.74 (s, 3H), 4.30 (m, 3H), 5.87 (s, 1H), 6.71 (dd, $J1$ = 2.6, $J2$ = 8.8, 1H), 6.95 (s, 1H), 7.32 (m, 9H), 7.57 (m, 3H), 7.88 (m, 2H);

^{13}C NMR ($CDCl_3$, 100 MHz) δ (ppm): 22.7, 34.7, 45.7, 46.5, 55.4, 59.7, 66.6, 83.8, 108.7, 114.5, 118.8, 120.0, 124.9, 125.7, 127.0, 127.6, 129.1, 129.5, 135.3, 136.7, 140.6, 140.7, 143.5, 143.6, 153.6, 156.4, 168.8;

Enantiomeric excess: 76%, determined by HPLC (Daicel Chirapak IA-H, hexane/isopropanol = 70/30, flow rate 1.0 mL/min, T = 30 °C, 254 nm): t_R = 14.16 min(minor), t_R = 22.71 min(major); IR (KBr): 2921, 2858, 1740, 1709, 1652, 1507, 1362, 1248, 1166, 831, 755, 699; HRMS exact mass calcd for $(C_{34}H_{30}N_2O_4Se + H)^+$ requires m/z 611.1449, found 611.1443.

(9*H*-Fluoren-9-yl)methyl 8-acetyl-6-bromo-3a-(phenylselenyl)-3,3a,8,8a-tetrahydropyrrolo[2,3-b]indole-1(2H)-carboxylate (4e)

46 mg (Flash column chromatography eluent, petroleum ether/ethyl acetate = 6/1–4/1), 70% yield,

^{1}H NMR (D-DMSO, 400 MHz) δ (ppm): 2.28 (m, 4H), 2.70 (m, 2H), 3.51 (m, 1H), 4.35 (m, 3H), 5.95 (s, 1H), 7.30 (m, 12H), 7.55 (m, 2H), 7.85 (m, 2H);

^{13}C NMR (D-DMSO, 100 MHz) δ (ppm): 22.9, 34.6, 45.7, 46.6, 54.8, 66.6, 84.0, 120.0, 120.1, 121.3, 124.9, 135.3, 125.6, 127.0, 127.2, 127.6, 129.1, 129.6, 132.2, 136.7, 140.0, 140.1, 143.0, 143.4, 143.5, 153.6, 169.6;

Enantiomeric excess: 86%, determined by HPLC (Daicel Chirapak IA-H, hexane/isopropanol = 70/30, flow rate 1.0 mL/min, T = 30 °C, 254 nm): t_R = 11.93 min(minor), t_R = 25.89 min(major); IR (KBr): 2921, 2858, 1740, 1709, 1652, 1507, 1362, 1248, 1166, 831, 755, 699; HRMS exact mass

calcd for ($C_{33}H_{27}BrN_2O_3Se$ + H)$^+$ requires *m*/*z* 659.0449, found 659.0443.

(9*H*-Fluoren-9-yl)methyl 8-acetyl-6-chloro-3a-(phenylselenyl)-3,3a,8,8a-tetrahydropyrrolo[2,3-*b*]indole-1(2*H*)-carboxylate (4f)

52 mg (Flash column chromatography eluent, petroleum ether/ethyl acetate = 6/1–4/1), 85% yield, ^{1}H NMR ($CDCl_3$, 400 MHz) δ (ppm): 2.28 (m, 4H), 2.67 (m, 2H), 3.52 (m, 1H), 4.35 (m, 3H), 5.97 (s, 1H), 7.40 (m, 11H), 7.58 (m, 3H), 7.88 (m, 2H);

^{13}C NMR ($CDCl_3$, 100 MHz) δ (ppm): 22.8, 34.7, 45.7, 46.6, 66.6, 84.2, 117.3 120.0, 124.3, 124.9, 125.3, 125.4, 127.0, 127.6, 129.1, 129.6, 131.8, 132.5, 132.8, 136.6, 140.7, 142.8, 143.4, 143.6, 153.6, 169.6;

Enantiomeric excess: 89%, determined by HPLC (Daicel Chirapak IA-H, hexane/isopropanol = 70/30, flow rate 1.0 mL/min, *T* = 30 °C, 254 nm): t_R = 9.61 min(minor), t_R = 20.72 min(major); IR (KBr): 2921, 2858, 1740, 1709, 1652, 1507, 1362, 1248, 1166, 831, 755, 699; HRMS exact mass calcd for ($C_{33}H_{27}ClN_2O_3Se$ + H)$^+$ requires *m*/*z* 615.0954, found 615.0948.

(9*H*-Fluoren-9-yl)methyl 8-acetyl-3a-((4-chlorophenyl)selenyl)-3,3a,8,8a-tetrahydropyrrolo[2,3-*b*]indole-1(2*H*)-carboxylate (4g)

43 mg (Flash column chromatography eluent, petroleum ether/ethyl acetate = 6/1–4/1), 71% yield, ^{1}H NMR ($CDCl_3$, 400 MHz) δ (ppm): 2.28 (m, 4H), 2.73 (m, 2H), 3.54 (m, 1H), 4.30 (m, 3H), 5.97 (s, 1H), 7.35 (m, 10H), 7.58 (m, 3H), 7.88 (m, 3H);

^{13}C NMR ($CDCl_3$, 100 MHz) δ (ppm): 22.8, 34.6, 45.8, 46.5, 55.8, 66.7, 83.9, 119.0, 120.0, 122.9, 123.8, 124.9, 125.3, 127.0, 127.6, 128.1, 128.7, 129.1, 129.6, 132.6, 134.2, 136.7, 140.7, 143.4, 143.6, 153.6, 169.1;

Enantiomeric excess: 83%, determined by HPLC (Daicel Chirapak IA-H, hexane/isopropanol = 70/30, flow rate 1.0 mL/min, *T* = 30 °C, 254 nm): t_R = 12.12 min(minor), t_R = 28.62 min(major); IR (KBr): 2921, 2858, 1740, 1709, 1652, 1507, 1362, 1248, 1166, 831, 755, 699; HRMS exact mass calcd for ($C_{33}H_{27}ClN_2O_3Se$ + H)$^+$ requires *m*/*z* 615.0954, found 615.0959.

(9*H*-Fluoren-9-yl)methyl 8-acetyl-3a-((4-fluorophenyl)selenyl)-3,3a,8,8a-tetrahydropyrrolo[2,3-*b*]indole-1(2*H*)-carboxylate (4h)

45 mg (Flash column chromatography eluent, petroleum ether/ethyl acetate = 6/1–4/1), 74% yield,

^{1}H NMR ($CDCl_3$, 400 MHz) δ (ppm): 2.28 (m, 4H), 2.73 (m, 2H), 3.54 (m, 1H), 4.30 (m, 3H), 5.97 (s, 1H), 7.35 (m, 10H), 7.58 (m, 3H), 7.68 (m, 3H), 8.02(m, 1H);

^{13}C NMR ($CDCl_3$, 100 MHz) δ (ppm): 22.8, 34.6, 45.8, 46.5, 55.8, 66.7, 83.9, 119.0, 120.0, 122.9, 123.8, 124.9, 125.3, 127.0, 127.6, 128.1, 128.7, 129.1, 129.6, 132.6, 134.2, 136.7, 140.7, 143.4, 143.6, 153.6, 169.1;

Enantiomeric excess: 86%, determined by HPLC (Daicel Chirapak IA-H, hexane/isopropanol = 70/30, flow rate 1.0 mL/min, T = 30 °C, 254 nm): t_R = 11.83 min(minor), t_R = 25.17 min(major); IR (KBr): 2921, 2858, 1740, 1709, 1652, 1507, 1362, 1248, 1166, 831, 755, 699; HRMS exact mass calcd for $(C_{33}H_{27}FN_2O_3S$ e+ $H)^+$ requires m/z 599.1244, found 599.1240.

(9*H*-Fluoren-9-yl)methyl 8-acetyl-3a-((4-ethoxyphenyl)selenyl)-3,3a,8,8a-tetrahydropyrrolo[2,3-*b*]indole-1(2*H*)-carboxylate (4i)

40 mg (Flash column chromatography eluent, petroleum ether/ethyl acetate = 6/1–4/1), 65% yield,

^{1}H NMR ($CDCl_3$, 400 MHz) δ (ppm): 1.35 (t, J = 6.96, 3H), 2.40 (m, 5H), 2.73 (m, 1H), 3.91 (m, 1H), 3.94 (q, J = 6.96, 2H), 4.30 (m, 3H), 5.97 (s, 1H), 7.35 (m, 10H), 7.58 (m, 3H), 7.88 (m, 3H);

^{13}C NMR ($CDCl_3$, 100 MHz) δ (ppm): 14.6, 26.9 ,46.0, 47.1, 63.5, 67.4, 115.3, 116.6, 118.8, 120.0, 123.4, 123.5, 124.6, 124.9, 127.0, 127.1, 127.7, 129.1, 132.7, 134.2, 138.6, 141.3, 142.6, 143.7, 154.4, 160.2, 168.1;

Enantiomeric excess: 84%, determined by HPLC (Daicel Chirapak IA-H, hexane/isopropanol = 70/30, flow rate 1.0 mL/min, T = 30 °C, 254 nm): t_R = 10.50 min(minor), t_R = 20.84 min(major); IR (KBr): 2921, 2858, 1740, 1709, 1652, 1507, 1362, 1248, 1166, 831, 755, 699; HRMS exact mass calcd for $(C_{35}H_{32}N_2O_4Se + H)^+$ requires m/z 625.1500, found 625.1496.

Directly catalytic asymmetric synthesis of (3aS,8aR)-(9*H*-fluoren-9-yl)methyl 8-acetyl-3a-(phenylselenyl)-3,3a,8,8a-tetrahydropyrrolo[2,3-*b*]indole-1(2*H*)-carboxylate (4a).

A mixture of *N*-phenylselenophthalimide (*N*-PSP) (3 mmol, 902 mg), catalyst **3b** (0.1 mmol, 70 mg), Fmoc-Ac-tryptamine (2 mmol, 850 mg), and 5 Å molecular sieves (500 mg) was added to a Schlenk tube, and the tube was flushed with argon gas three times. Then, the DCE (20 mL) was added at 0 °C. The reaction mixture was stirred at 0 °C until the reaction was complete (the reaction time was 5 d, monitored by TLC). The reaction mixture was extracted with CH_2Cl_2 (3 × 20 mL). The combined organic layers were dried over anhydrous Na_2SO_4, and concentrated in vacuo. The

residue was purified by flash column chromatography (petroleum ether:ethyl acetate = 6:1–4:1) on silica gel to give the product **4a** (930 mg, 80% yield, 82% ee). After a single recrystallization from methanol, the product **4a** was obtained in 50% yield and with 97% ee.

Synthesis of (3a*S*,8a*R*)-(9*H*-fluoren-9-yl)methyl 8-acetyl-3a-hydroxy-3,3a,8,8a-tetrahydropyrrolo[2,3-*b*]indole-1(2*H*)-carboxylate (5).

To a solution of **4a** (500 mg, 0.86 mmol) and K_2CO_3 (469 mg, 3.4 mmol) in DCM (30 mL) was added wet MCPBA (70%) (1.0 g, 5 mmol) at room temperature. Afterward, the reaction was stirred at room temperature for 10 mins, brine was added, and then the crude mixture was extracted with CH_2Cl_2 (2 × 20 mL). The combined organic layers were washed with brine and dried over anhydrous Na_2SO_4. After concentration under reduced pressure, the residue obtained was purified by column chromatography (petroleum ether:ethyl acetate = 1:1) to afford the title compound **5** (360 mg, 95%).

^{1}H NMR ($CDCl_3$, 400 MHz) δ (ppm): 2.64 (m, 6H), 3.61 (m, 1H), 4.20 (m, 1H), 4.49 (m, 2H), 6.05 (s, 1H), 7.2(m, 1H), 7.45 (m, 6H), 7.58 (m, 2H), 7.88 (m, 2H), 8.02 (m, 1H);

^{13}C NMR ($CDCl_3$, 100 MHz) δ (ppm): 23.4, 39.6, 46.4, 47.2, 67.5, 84.6, 119.1, 120.0, 123.2, 123.5, 124.8, 125.2, 127.1, 127.2, 127.8, 127.9, 130.8, 134.2, 141.3, 141.4, 142.4, 143.5, 154.4, 168.1, 170.8;

IR (KBr): 2930, 2854, 1745, 1740, 1652, 1507, 1362, 1248, 1166, 852, 840, 689; HRMS exact mass calcd for $(C_{27}H_{24}N_2O_4 + H)^+$ requires *m*/*z* 441.1809, found 441.1811.

X-ray single crystal data for 4a

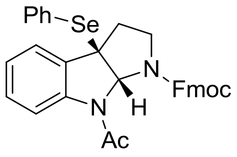

4a

(3a*R*,8a*S*)-(9*H*-fluoren-9-yl)methyl 8-acetyl-3a-(phenylselanyl)-3,3a,8,8a-tetrahydropyrrolo[2,3-*b*]indole-1(2*H*)-carboxylate

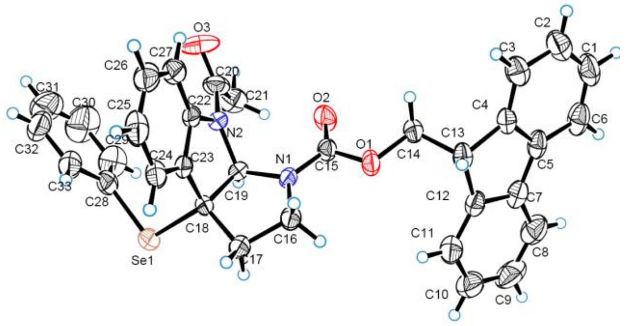

```
Bond precision:  C-C = 0.0059 A          Wavelength=0.71073

Cell:             a=9.3282(3)      b=16.0584(6)      c=18.4981(6)
                  alpha=90         beta=90           gamma=90
Temperature:      291 K

                 Calculated                  Reported
Volume           2770.94(16)                 2770.94(16)
Space group      P 21 21 21                  P 21 21 21
Hall group       P 2ac 2ab                   ?
Moiety formula   C33 H28 N2 O3 Se            ?
Sum formula      C33 H28 N2 O3 Se            C33 H28 N2 O3 Se
Mr               579.53                      579.53
Dx,g cm-3        1.389                       1.389
Z                4                           4
Mu (mm-1)        1.392                       1.392
F000             1192.0                      1192.0
F000'            1192.07
h,k,lmax         10,18,21                    10,18,21
Nref             2672[ 4705]                 4695
Tmin,Tmax        0.571,0.641                 0.599,0.664
Tmin'            0.560

Correction method= MULTI-SCAN

Data completeness= 1.76/1.00       Theta(max)= 24.690

R(reflections)= 0.0419( 3828)      wR2(reflections)= 0.0735( 4695)

S = 1.024                  Npar= 353
```

SAMPLE INFORMATION

Sample Name:	se-Fmoc_mg100_psp-ia30%	Acquired By:	System
Sample Type:	Unknown	Date Acquired:	5/30/2011 11:40:34 AM
Vial:	1	Acq. Method:	wq1_30%
Injection #:	4	Date Processed:	1/14/2012 12:00:48 AM
Injection Volume:	20.00 ul	Channel Name:	2487Channel 1
Run Time:	70.00 Minutes	Sample Set Name:	

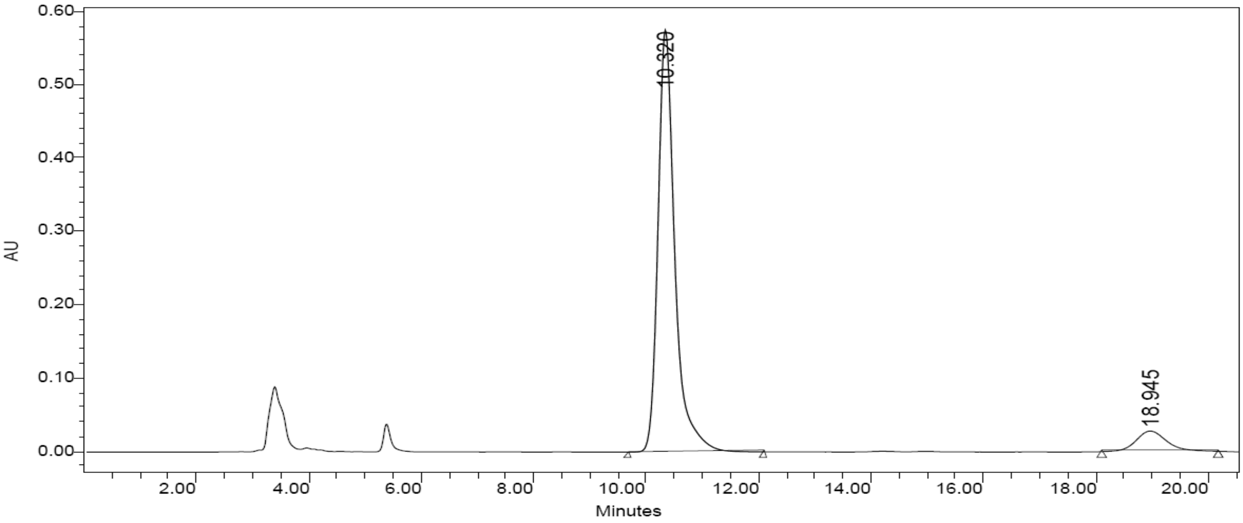

	RT (min)	Area (V*sec)	% Area	Height (V)	% Height
1	10.320	11992518	91.87	575216	95.37
2	18.945	1061698	8.13	27900	4.63

SAMPLE INFORMATION

Sample Name:	se-1crys-IA30%	Acquired By:	System
Sample Type:	Unknown	Date Acquired:	9/12/2011 8:52:09 PM
Vial:	1	Acq. Method:	wq1_30%
Injection #:	1	Date Processed:	4/10/2013 4:41:32 AM
Injection Volume:	20.00 ul	Channel Name:	2487Channel 1
Run Time:	70.00 Minutes	Sample Set Name:	

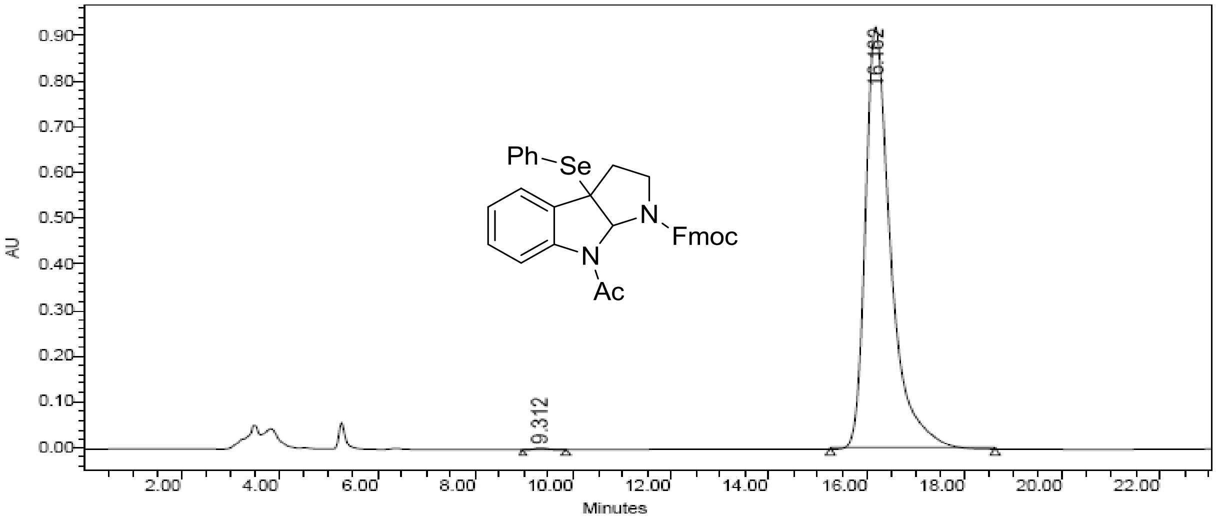

	RT (min)	Area (V*sec)	% Area	Height (V)	% Height
1	9.312	54714	0.16	2925	0.32
2	16.162	33277304	99.84	920049	99.68

SAMPLE INFORMATION

Sample Name:	se-s-p-5f_DL-IA30%	Acquired By:	System
Sample Type:	Unknown	Date Acquired:	1/14/2012 2:16:34 AM
Vial:	1	Acq. Method:	wq1_30%
Injection #:	7	Date Processed:	1/14/2012 4:14:27 AM
Injection Volume:	20.00 ul	Channel Name:	2487Channel 1
Run Time:	70.00 Minutes	Sample Set Name:	

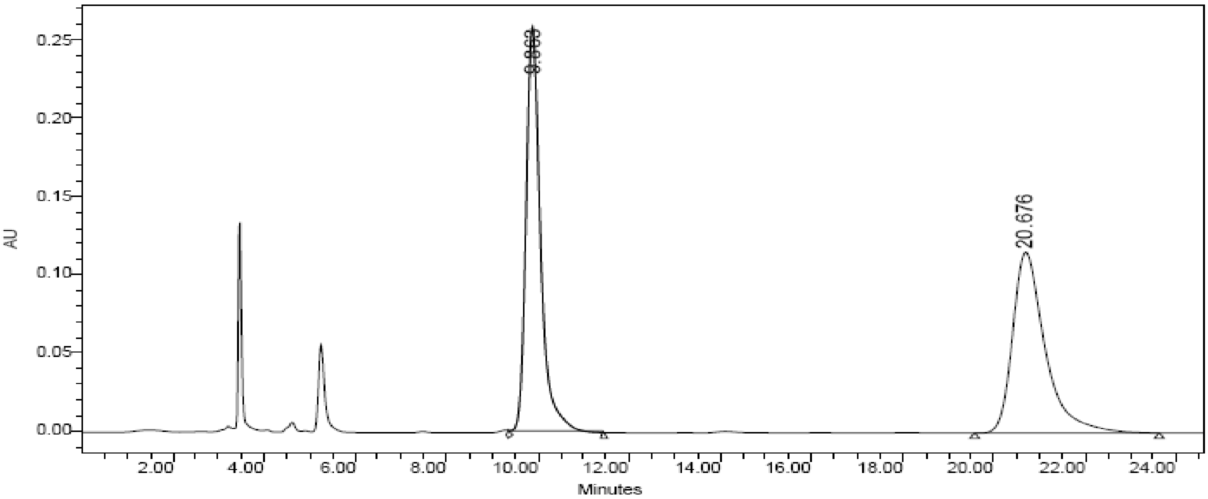

	RT (min)	Area (V*sec)	% Area	Height (V)	% Height
1	9.863	5771014	50.12	259579	69.20
2	20.676	5742579	49.88	115539	30.80

SAMPLE INFORMATION

Sample Name:	se-s-p-5f_p-IA30%	Acquired By:	System
Sample Type:	Unknown	Date Acquired:	1/14/2012 3:45:42 AM
Vial:	1	Acq. Method:	wq1_30%
Injection #:	10	Date Processed:	1/14/2012 4:12:47 AM
Injection Volume:	20.00 ul	Channel Name:	2487Channel 1
Run Time:	70.00 Minutes	Sample Set Name:	

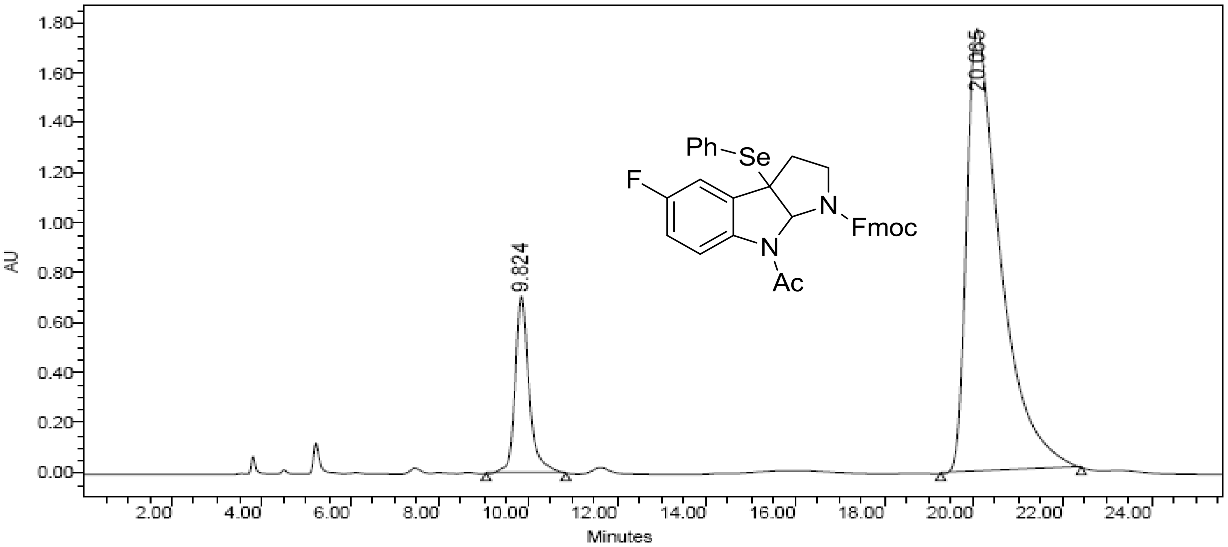

	RT (min)	Area (V*sec)	% Area	Height (V)	% Height
1	9.824	15793333	14.33	711276	28.61
2	20.065	94409210	85.67	1775170	71.39

SAMPLE INFORMATION

Sample Name:	se-5-5OMe_DL-IA30%	Acquired By:	System
Sample Type:	Unknown	Date Acquired:	9/9/2011 12:22:10 AM
Vial:	1	Acq. Method:	wq1_30%
Injection #:	3	Date Processed:	9/9/2011 12:53:51 AM
Injection Volume:	20.00 ul	Channel Name:	2487Channel 1
Run Time:	70.00 Minutes	Sample Set Name:	

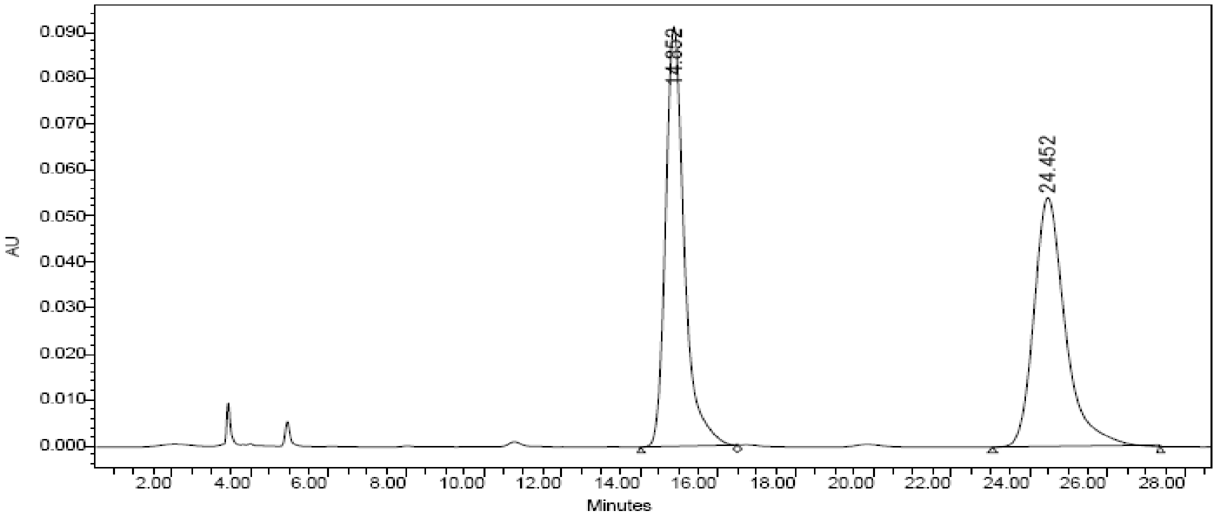

	RT (min)	Area (V*sec)	% Area	Height (V)	% Height
1	14.852	3064978	50.02	91281	62.75
2	24.452	3062855	49.98	54189	37.25

SAMPLE INFORMATION

Sample Name:	se-fmoc-5OMe_35-ia30%	Acquired By:	System
Sample Type:	Unknown	Date Acquired:	6/21/2011 11:05:18 AM
Vial:	1	Acq. Method:	wq1_30%
Injection #:	2	Date Processed:	1/13/2012 11:49:07 PM
Injection Volume:	20.00 ul	Channel Name:	2487Channel 1
Run Time:	70.00 Minutes	Sample Set Name:	

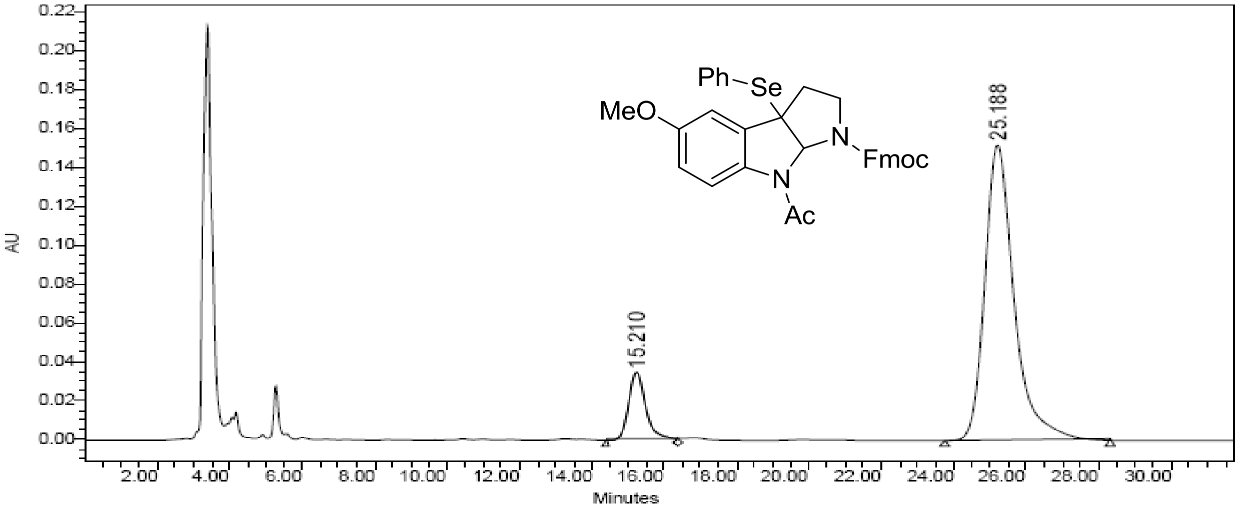

	RT (min)	Area (V*sec)	% Area	Height (V)	% Height
1	15.210	1171982	12.00	35057	18.72
2	25.188	8592794	88.00	152229	81.28

SAMPLE INFORMATION

Sample Name:	se-s-p-6cl_DL-IA30%	Acquired By:	System
Sample Type:	Unknown	Date Acquired:	1/14/2012 1:31:58 AM
Vial:	1	Acq. Method:	wq1_30%
Injection #:	5	Date Processed:	1/14/2012 1:58:13 AM
Injection Volume:	20.00 ul	Channel Name:	2487Channel 1
Run Time:	70.00 Minutes	Sample Set Name:	

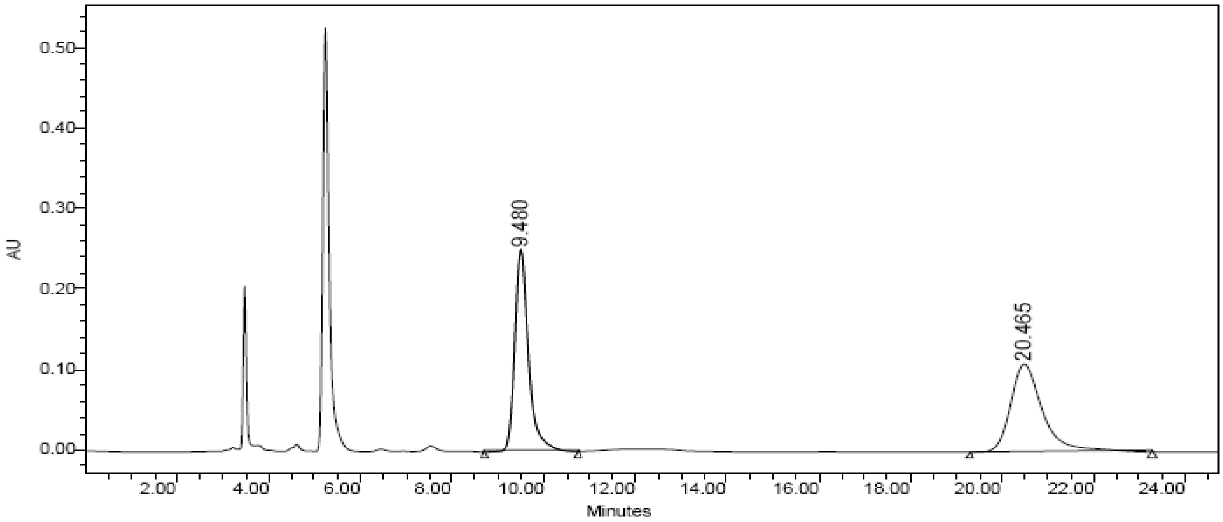

	RT (min)	Area (V*sec)	% Area	Height (V)	% Height
1	9.480	5259255	49.45	250859	69.63
2	20.465	5376157	50.55	109434	30.37

SAMPLE INFORMATION

Sample Name:	se-s-p-6CL2-IA30%	Acquired By:	System
Sample Type:	Unknown	Date Acquired:	1/7/2012 11:02:07 AM
Vial:	1	Acq. Method:	wq1_30%
Injection #:	1	Date Processed:	1/13/2012 11:49:07 PM
Injection Volume:	20.00 ul	Channel Name:	2487Channel 1
Run Time:	70.00 Minutes	Sample Set Name:	

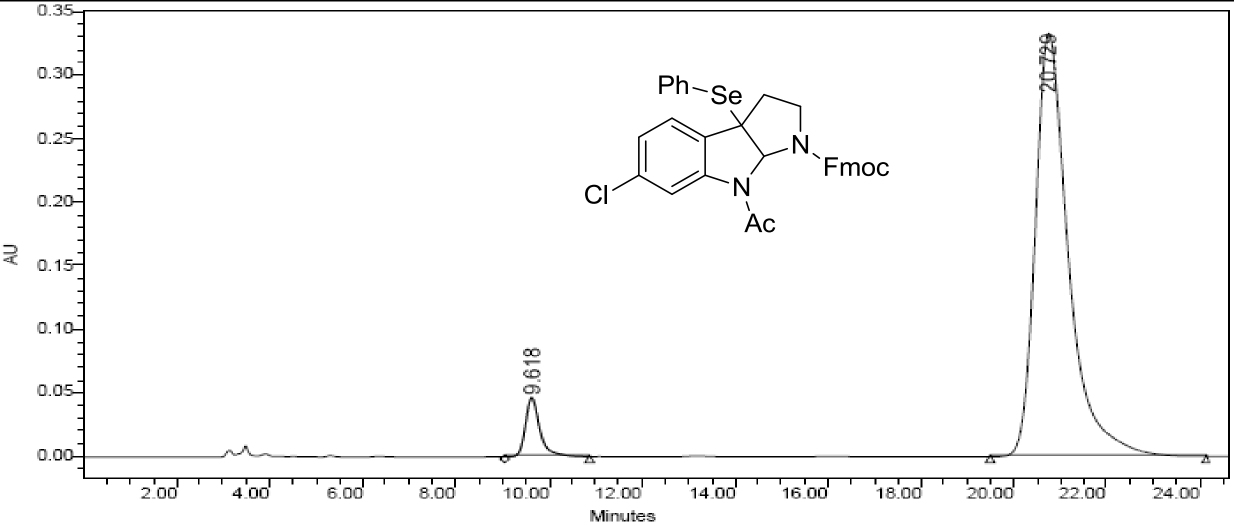

	RT (min)	Area (V*sec)	% Area	Height (V)	% Height
1	9.618	1013416	5.77	46608	12.26
2	20.729	16535420	94.23	333508	87.74

SAMPLE INFORMATION

Sample Name:	se-5-6f_DL-IA30%	Acquired By:	System
Sample Type:	Unknown	Date Acquired:	9/8/2011 5:57:08 PM
Vial:	1	Acq. Method:	wq1_30%
Injection #:	1	Date Processed:	9/8/2011 6:18:14 PM
Injection Volume:	20.00 ul	Channel Name:	2487Channel 1
Run Time:	70.00 Minutes	Sample Set Name:	

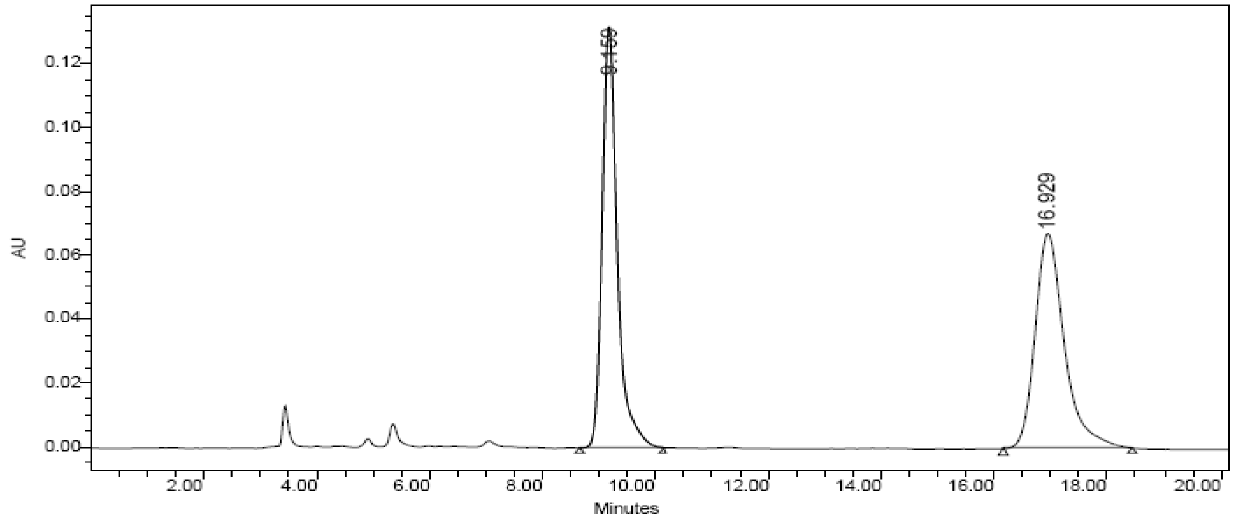

	RT (min)	Area (V*sec)	% Area	Height (V)	% Height
1	9.159	2459343	50.24	131894	66.22
2	16.929	2435914	49.76	67269	33.78

SAMPLE INFORMATION

Sample Name:	se-s-p-6f_ia30%	Acquired By:	System
Sample Type:	Unknown	Date Acquired:	12/28/2011 9:34:50 AM
Vial:	1	Acq. Method:	wq1_30%
Injection #:	1	Date Processed:	1/13/2012 11:49:07 PM
Injection Volume:	20.00 ul	Channel Name:	2487Channel 1
Run Time:	70.00 Minutes	Sample Set Name:	

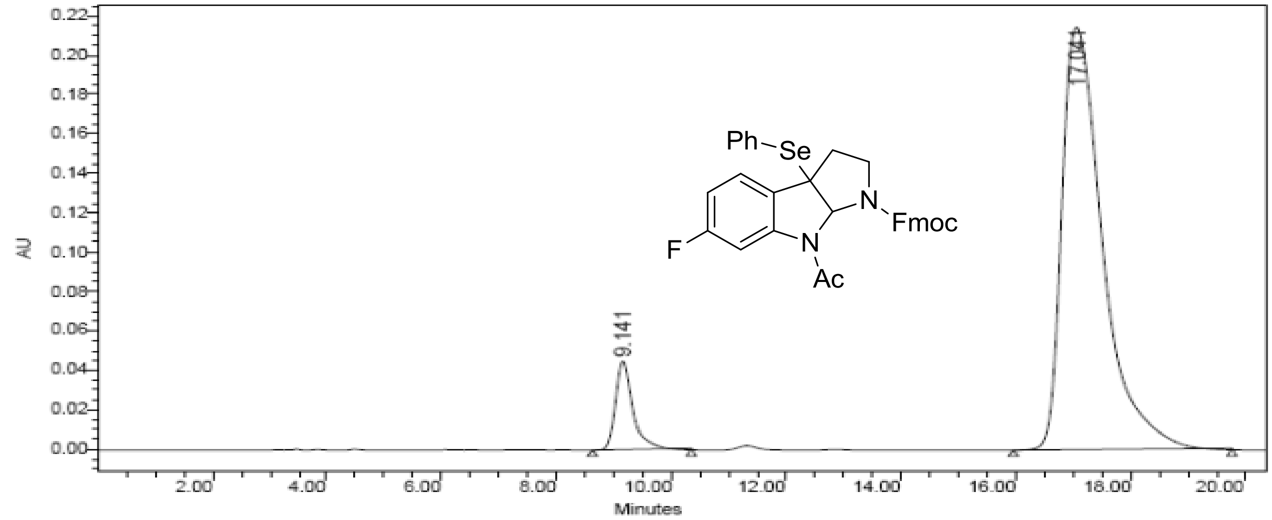

	RT (min)	Area (V*sec)	% Area	Height (V)	% Height
1	9.141	963902	8.31	45085	17.37
2	17.041	10638230	91.69	214448	82.63

SAMPLE INFORMATION

Sample Name:	se-5-6Br_DL-IA30%	Acquired By:	System
Sample Type:	Unknown	Date Acquired:	9/9/2011 1:21:23 AM
Vial:	1	Acq. Method:	wq1_30%
Injection #:	5	Date Processed:	9/9/2011 1:49:31 AM
Injection Volume:	20.00 ul	Channel Name:	2487Channel 1
Run Time:	70.00 Minutes	Sample Set Name:	

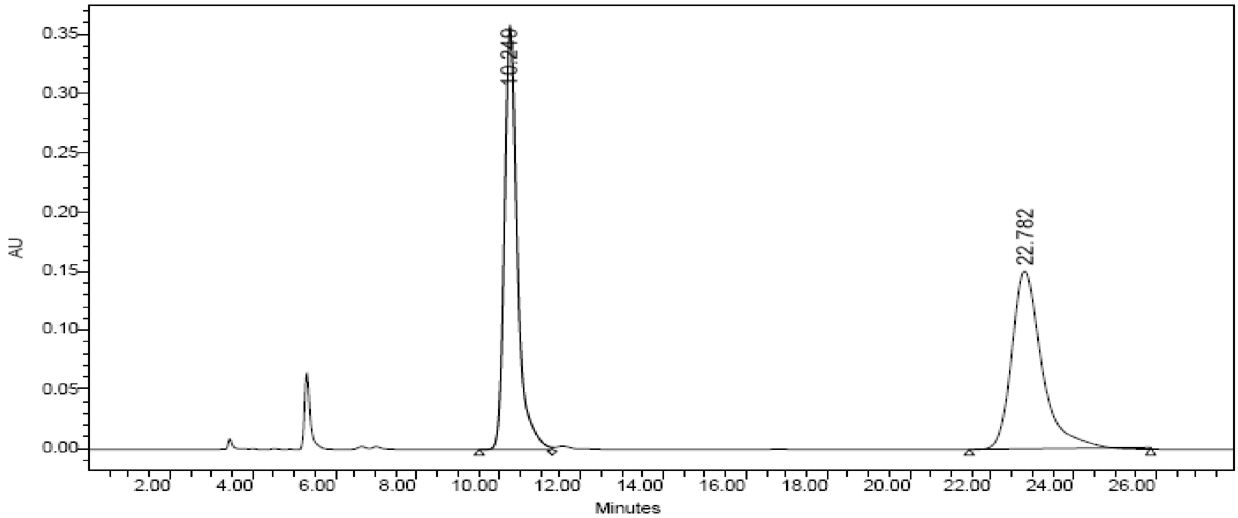

	RT (min)	Area (V*sec)	% Area	Height (V)	% Height
1	10.249	7745582	49.88	356691	70.29
2	22.782	7783560	50.12	150765	29.71

SAMPLE INFORMATION

Sample Name:	p-6b_ia30%	Acquired By:	System
Sample Type:	Unknown	Date Acquired:	10/30/2011 10:45:35 AM
Vial:	1	Acq. Method:	wq1_30%
Injection #:	1	Date Processed:	1/14/2012 12:49:54 AM
Injection Volume:	20.00 ul	Channel Name:	2487Channel 1
Run Time:	70.00 Minutes	Sample Set Name:	

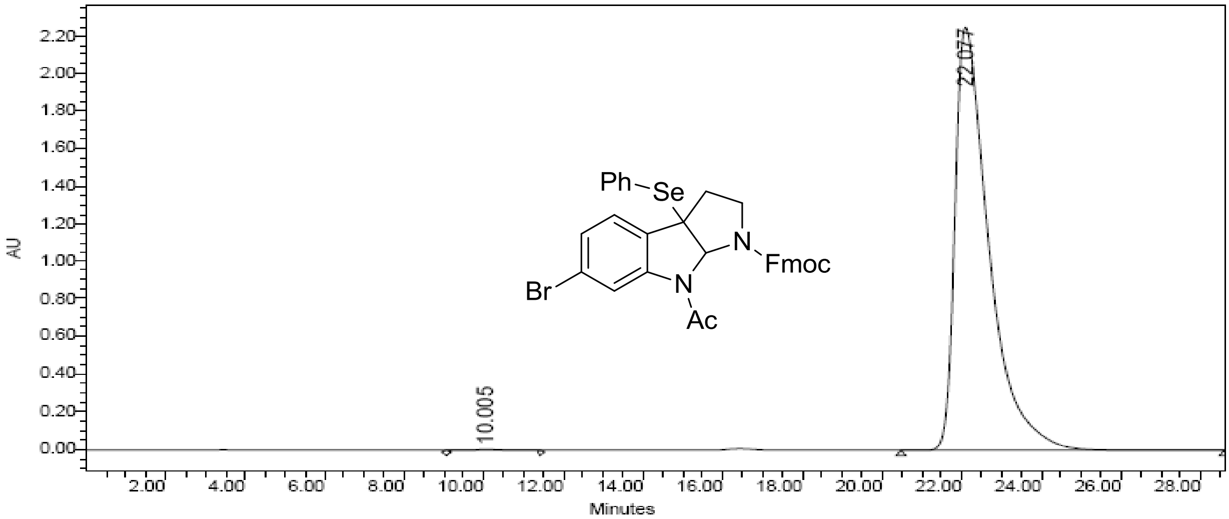

	RT (min)	Area (V*sec)	% Area	Height (V)	% Height
1	10.005	207023	0.15	4929	0.22
2	22.077	133490012	99.85	2251855	99.78

SAMPLE INFORMATION

Sample Name:	se-x-4f_ia30%dl	Acquired By:	System
Sample Type:	Unknown	Date Acquired:	4/3/2013 10:45:02 PM
Vial:	1	Acq. Method:	wq1_30%
Injection #:	5	Date Processed:	4/4/2013 1:15:47 AM
Injection Volume:	20.00 ul	Channel Name:	2487Channel 1
Run Time:	70.00 Minutes	Sample Set Name:	

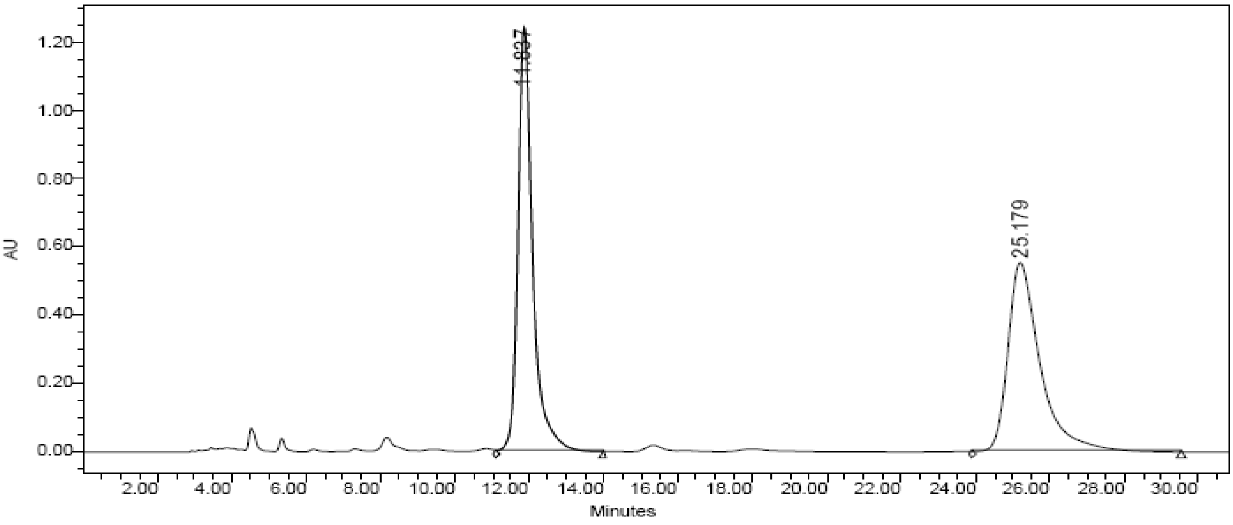

	RT (min)	Area (V*sec)	% Area	Height (V)	% Height
1	11.837	34506818	50.48	1245641	69.11
2	25.179	33854538	49.52	556723	30.89

SAMPLE INFORMATION

Sample Name:	se-x-2f_ia30%p	Acquired By:	System
Sample Type:	Unknown	Date Acquired:	4/3/2013 9:26:19 PM
Vial:	1	Acq. Method:	wq1_30%
Injection #:	2	Date Processed:	4/4/2013 1:18:37 AM
Injection Volume:	20.00 ul	Channel Name:	2487Channel 1
Run Time:	70.00 Minutes	Sample Set Name:	

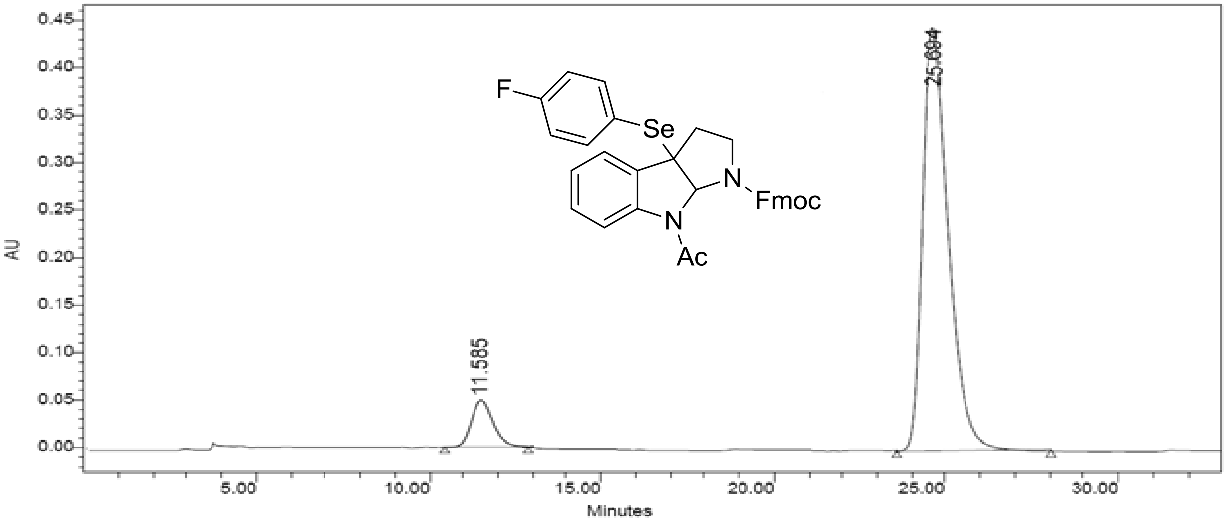

	RT (min)	Area (V*sec)	% Area	Height (V)	% Height
1	11.585	808371	8.52	41793	9.98
2	25.694	8677755	91.48	377147	90.02

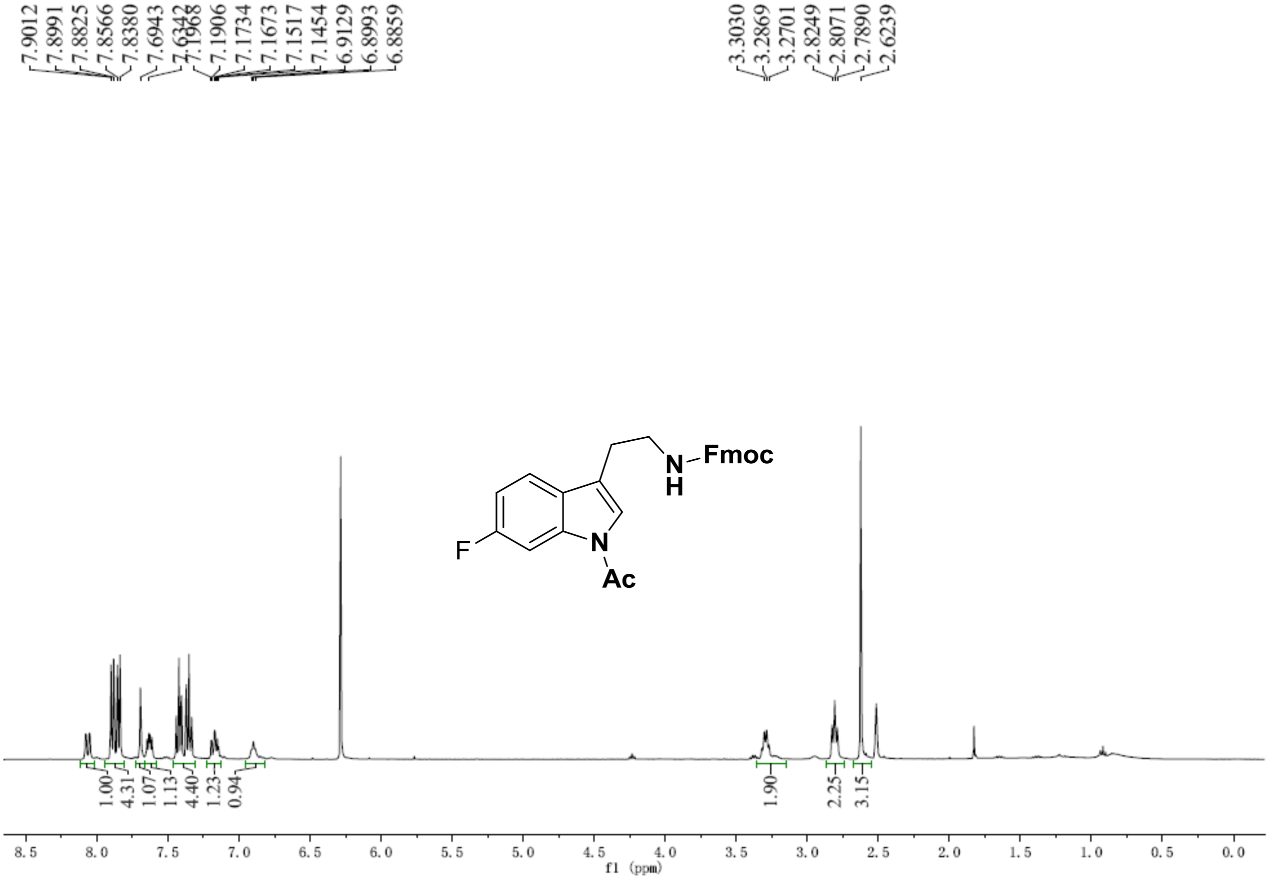
7.9012
7.8991
7.8825
7.8566
7.8380
7.6943
7.1906
7.1734
7.1673
7.1517
7.1454
6.9129
6.8993
6.8859
3.3030
3.2869
3.2701
2.8249
2.8071
2.7890
2.6239
N
H
Fmoc
F
N
Ac
1.00
4.31
1.07
1.13
4.40
1.23
0.94
1.90
2.25
3.15
f1 (ppm)

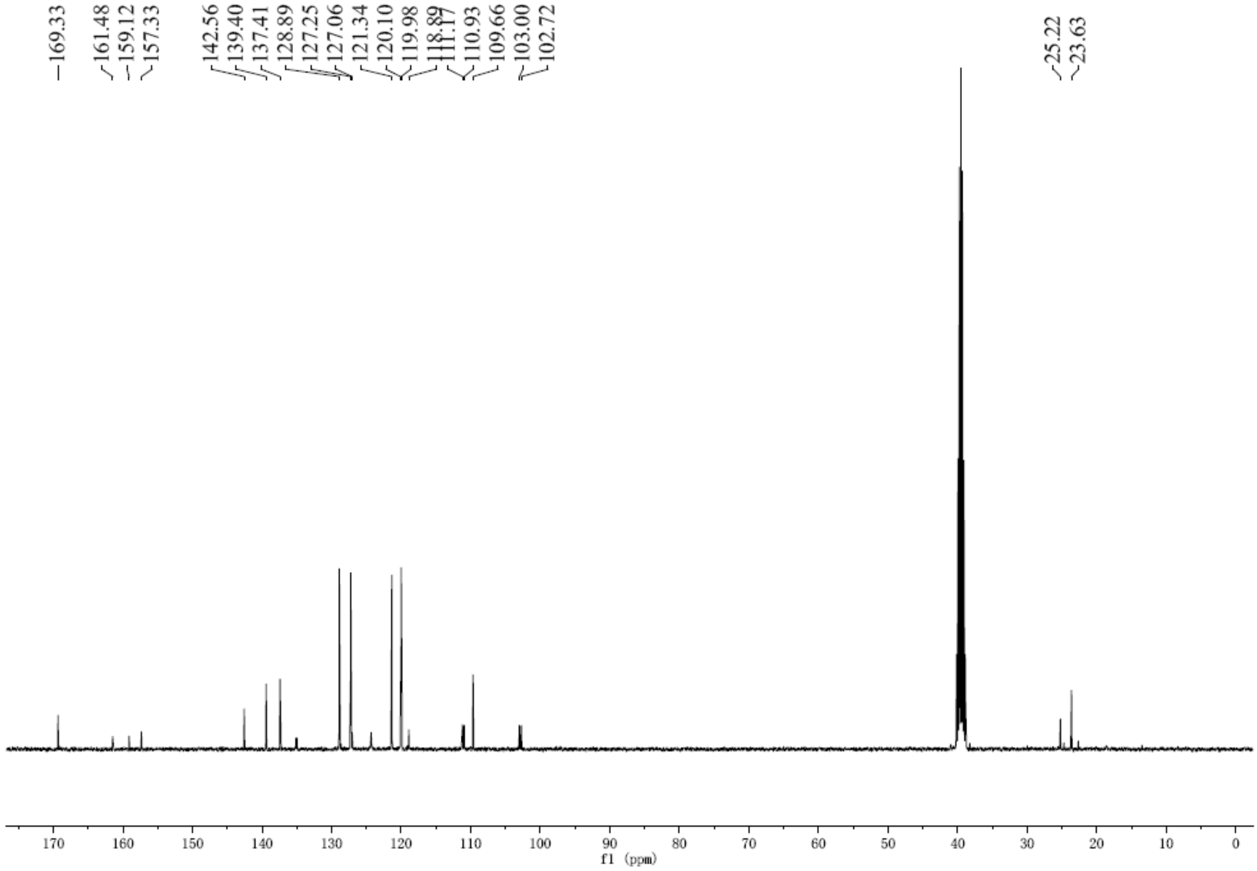
169.33
161.48
159.12
157.33
142.56
139.40
137.41
128.89
127.25
127.06
121.34
120.10
119.98
118.89
111.17
110.93
109.66
103.00
102.72
25.22
23.63
f1 (ppm)

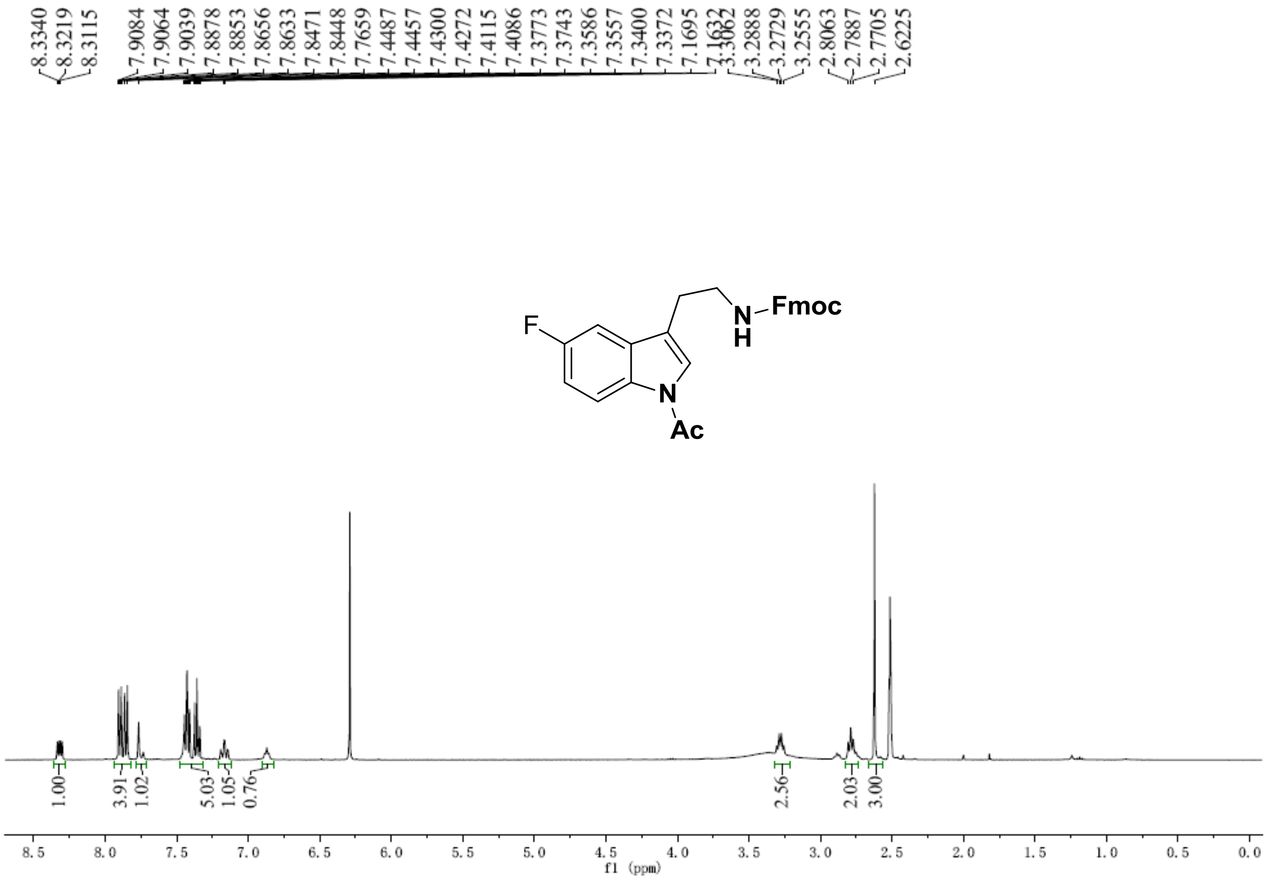
8.3340
8.3219
8.3115
7.9084
7.9064
7.9039
7.8878
7.8853
7.8656
7.8633
7.8471
7.8448
7.7659
7.4487
7.4457
7.4300
7.4272
7.4115
7.4086
7.3773
7.3743
7.3586
7.3557
7.3400
7.3372
7.1695
7.1632
3.3062
3.2888
3.2729
3.2555
2.8063
2.7887
2.7705
2.6225
F
N
H
Fmoc
N
Ac
1.00
3.91
1.02
5.03
1.05
0.76
2.56
2.03
3.00
8.5 8.0 7.5 7.0 6.5 6.0 5.5 5.0 4.5 4.0 3.5 3.0 2.5 2.0 1.5 1.0 0.5 0.0
f1 (ppm)

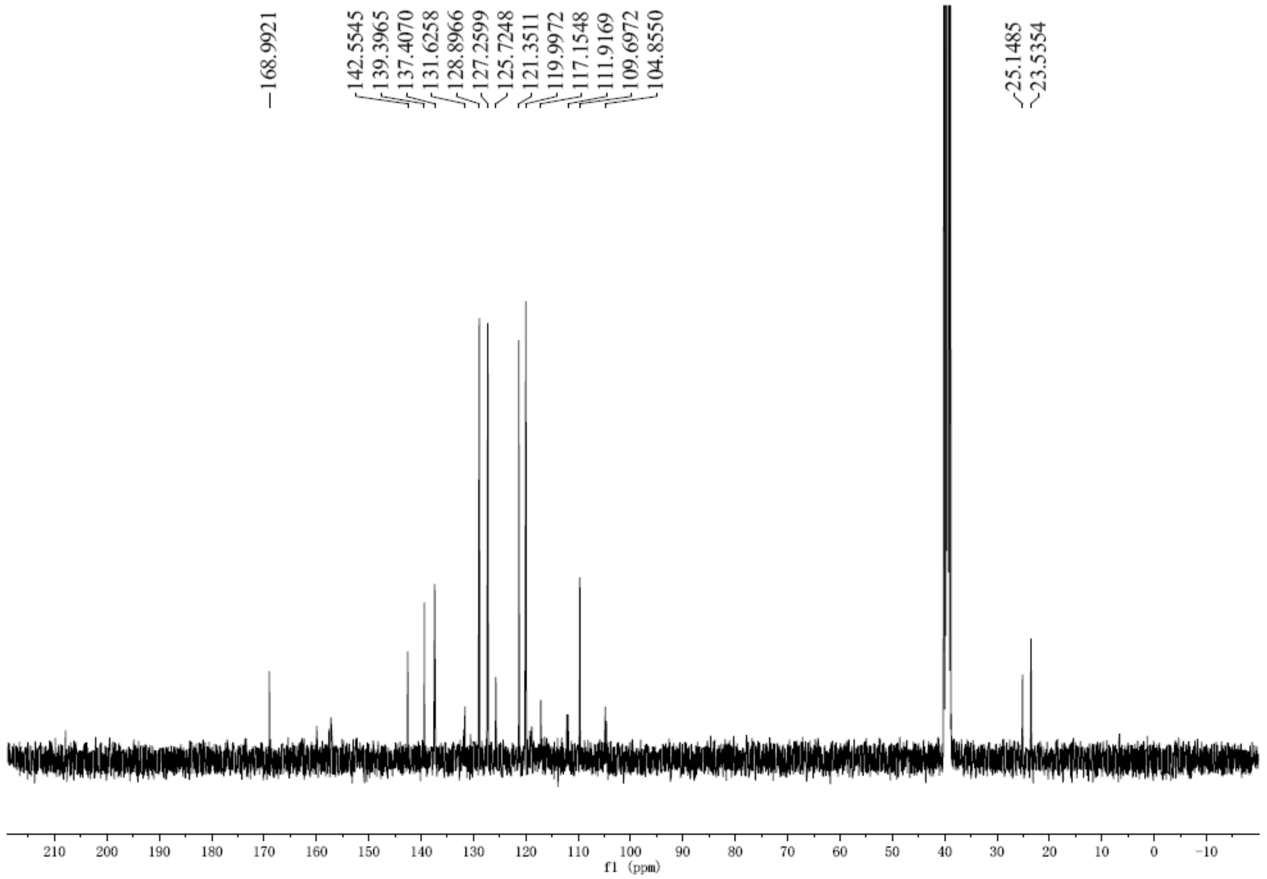
168.9921
142.5545
139.3965
137.4070
131.6258
128.8966
127.2599
125.7248
121.3511
119.9972
117.1548
111.9169
109.6972
104.8550
25.1485
23.5354
210 200 190 180 170 160 150 140 130 120 110 100 90 80 70 60 50 40 30 20 10 0 -10
f1 (ppm)

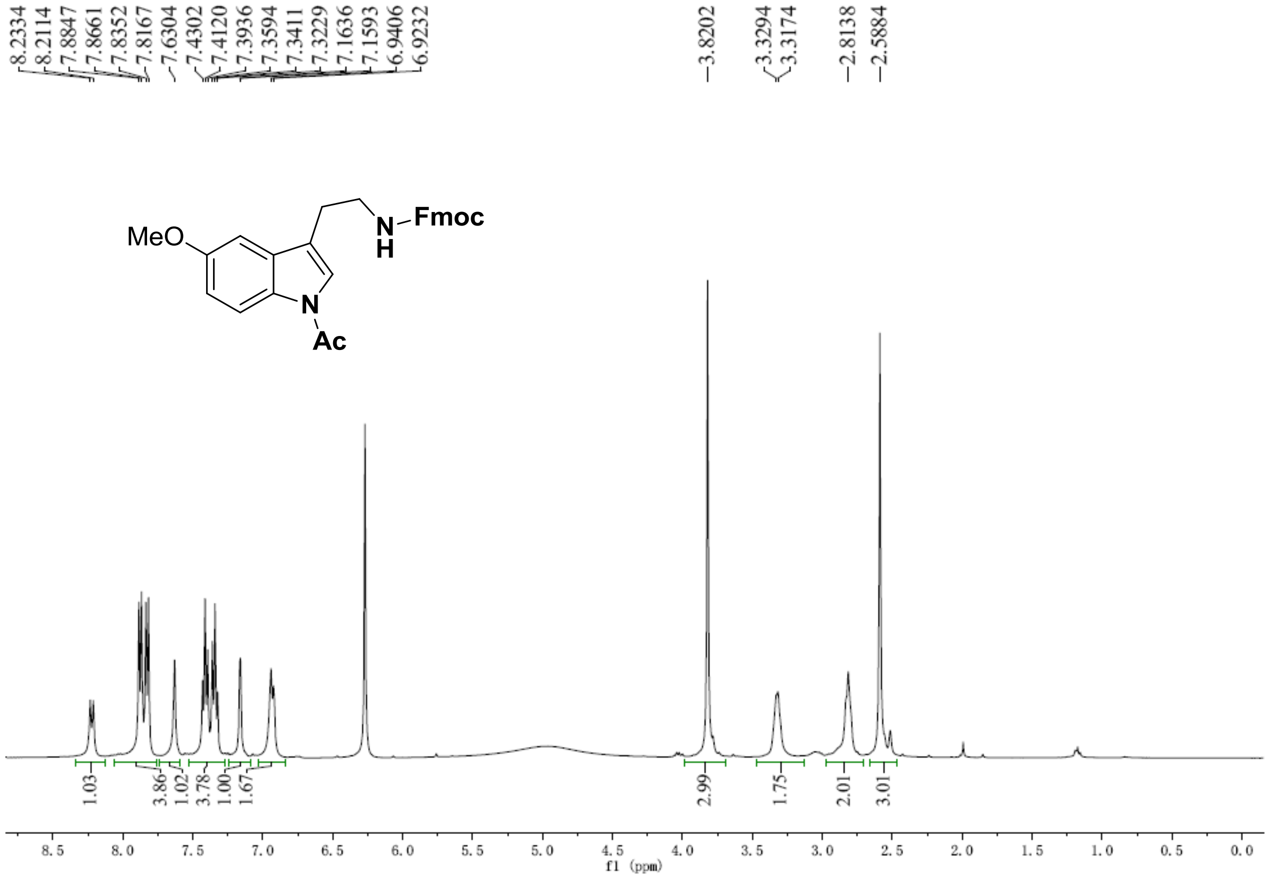
8.2334
8.2114
7.8847
7.8661
7.8352
7.8167
7.6304
7.4302
7.4120
7.3936
7.3594
7.3411
7.3229
7.1636
7.1593
6.9406
6.9232
3.8202
3.3294
3.3174
2.8138
2.5884
MeO
N
H
Fmoc
N
Ac
1.03
3.86
1.02
3.78
1.00
1.67
2.99
1.75
2.01
3.01
8.5 8.0 7.5 7.0 6.5 6.0 5.5 5.0 4.5 4.0 3.5 3.0 2.5 2.0 1.5 1.0 0.5 0.0
f1 (ppm)

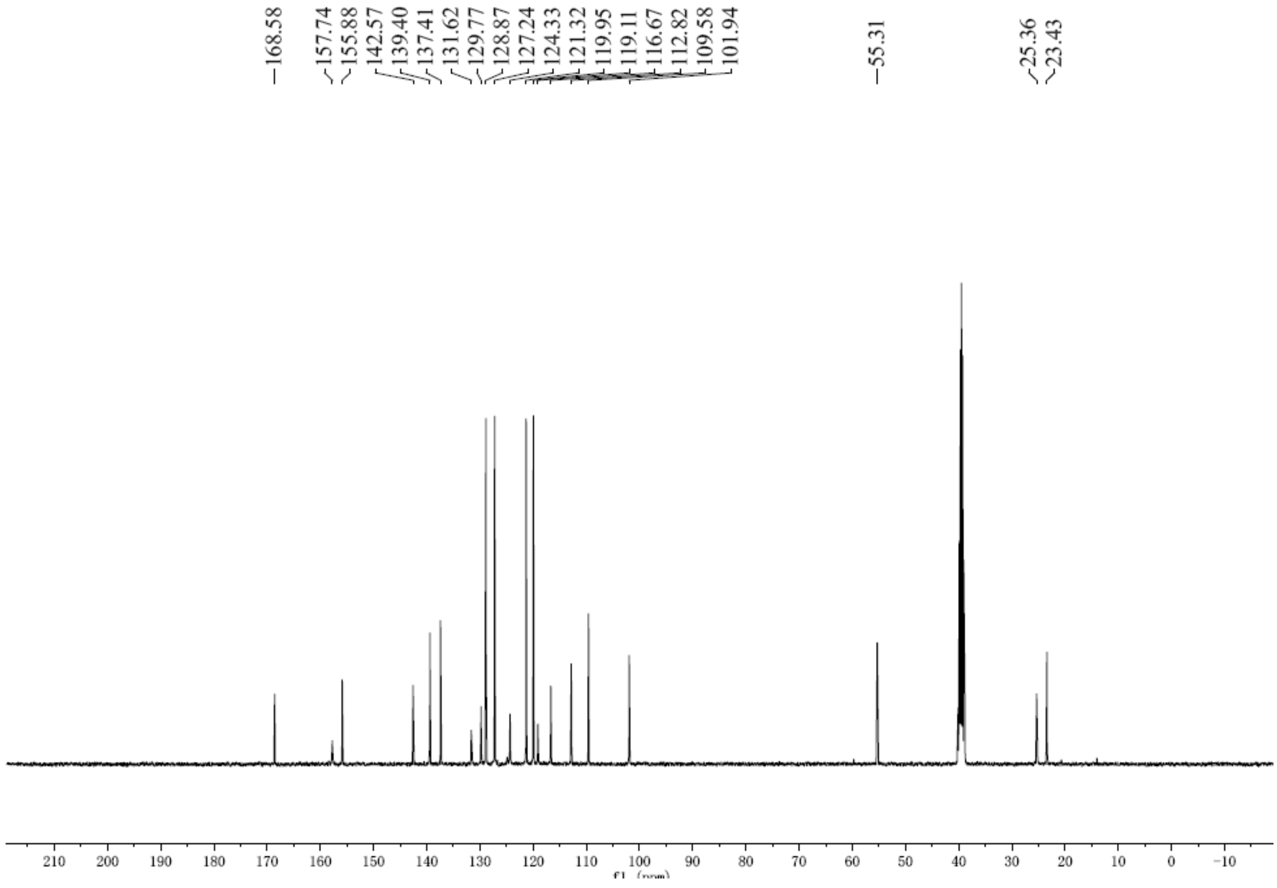
168.58
157.74
155.88
142.57
139.40
137.41
131.62
129.77
128.87
127.24
124.33
121.32
119.95
119.11
116.67
112.82
109.58
101.94
55.31
25.36
23.43
210 200 190 180 170 160 150 140 130 120 110 100 90 80 70 60 50 40 30 20 10 0 -10

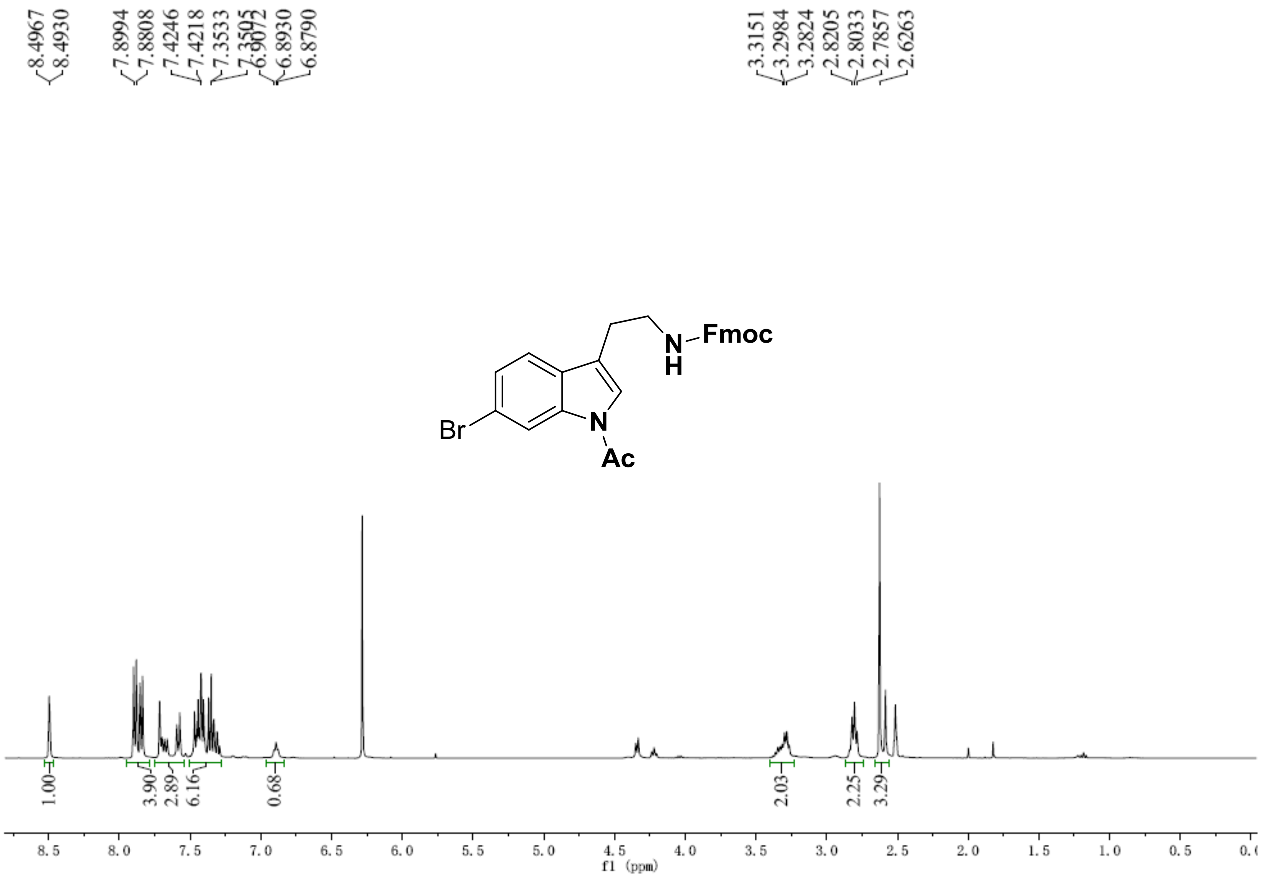
8.4967
8.4930
7.8994
7.8808
7.4246
7.4218
7.3533
7.3505
6.9072
6.8930
6.8790
3.3151
3.2984
3.2824
2.8205
2.8033
2.7857
2.6263
N-Fmoc
H
Br
N
Ac
1.00
3.90
2.89
6.16
0.68
2.03
2.25
3.29
f1 (ppm)

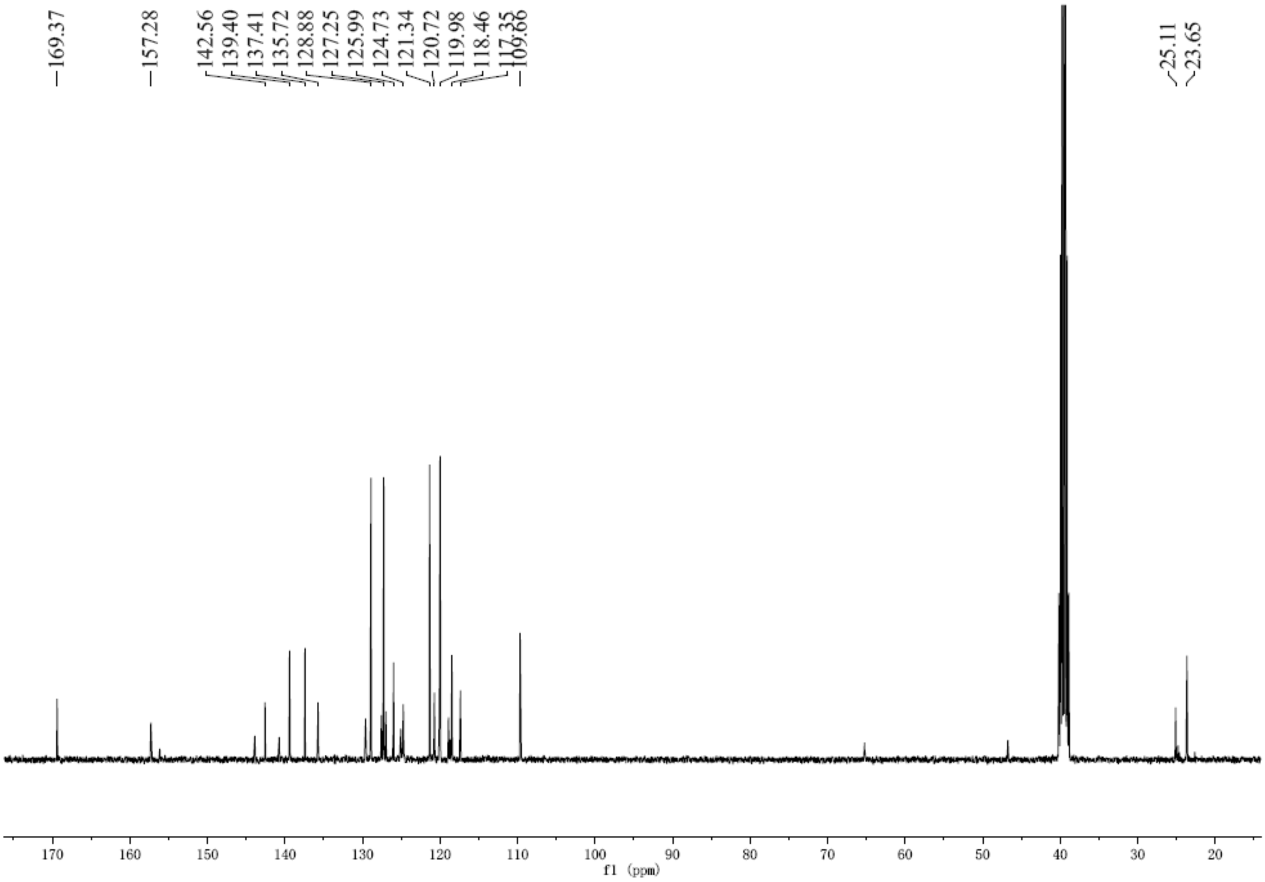
169.37
157.28
142.56
139.40
137.41
135.72
128.88
127.25
125.99
124.73
121.34
120.72
119.98
118.46
117.35
109.66
25.11
23.65
f1 (ppm)

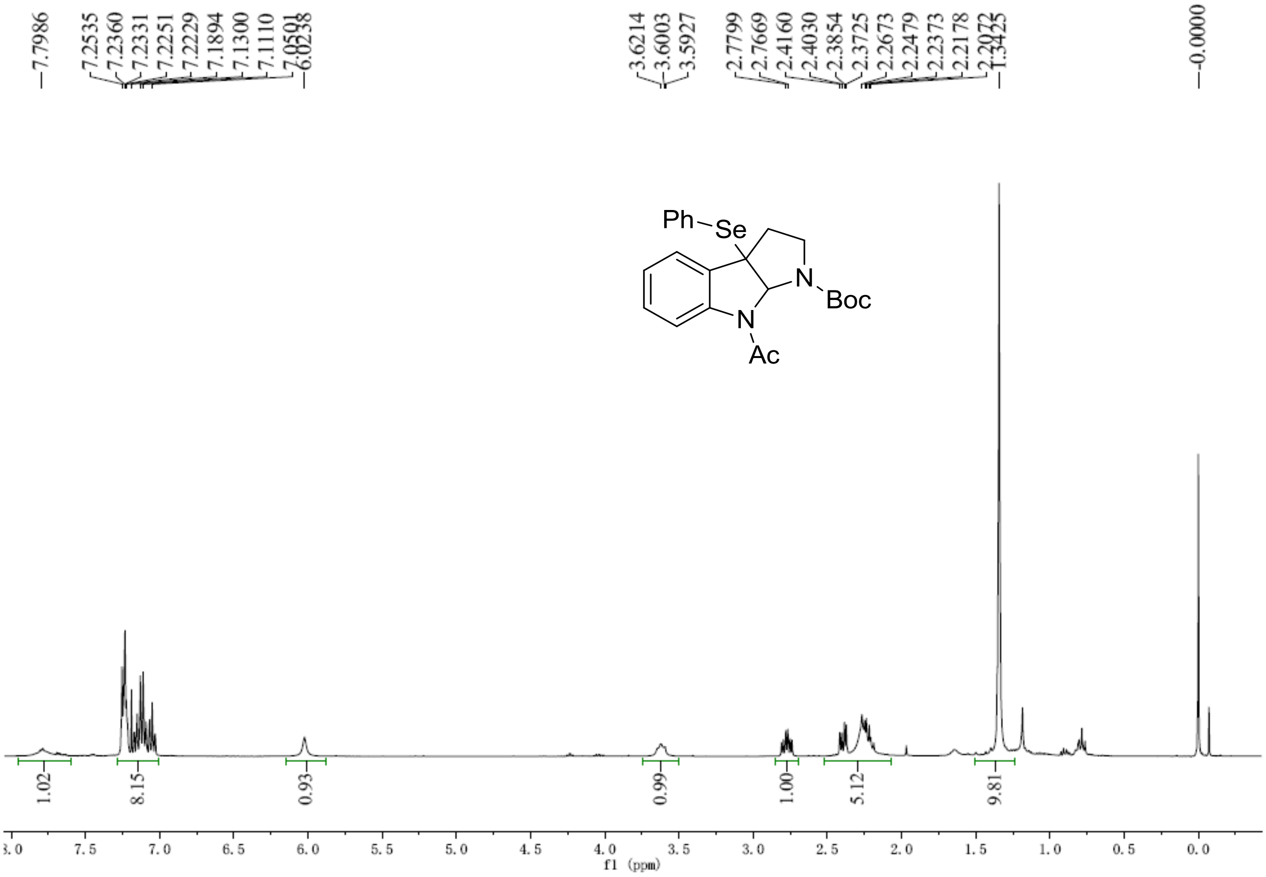
7.7986
7.2535
7.2360
7.2331
7.2251
7.2229
7.1894
7.1300
7.1110
7.0501
6.0238
3.6214
3.6003
3.5927
2.7799
2.7669
2.4160
2.4030
2.3854
2.3725
2.2673
2.2479
2.2373
2.2178
2.2072
1.3425
0.0000
Ph
Se
N
Boc
N
Ac
1.02
8.15
0.93
0.99
1.00
5.12
9.81
f1 (ppm)

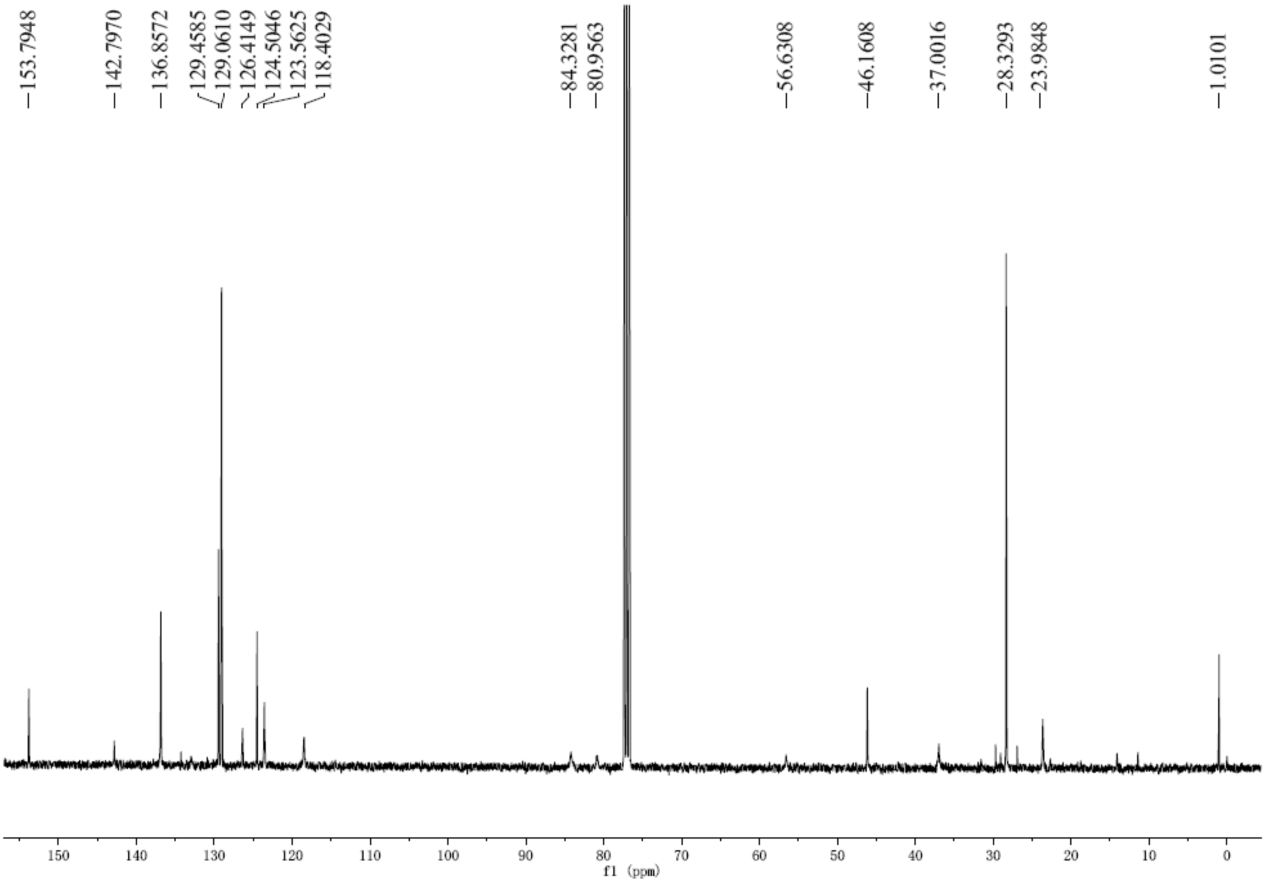
153.7948
142.7970
136.8572
129.4585
129.0610
126.4149
124.5046
123.5625
118.4029
84.3281
80.9563
56.6308
46.1608
37.0016
28.3293
23.9848
1.0101
f1 (ppm)

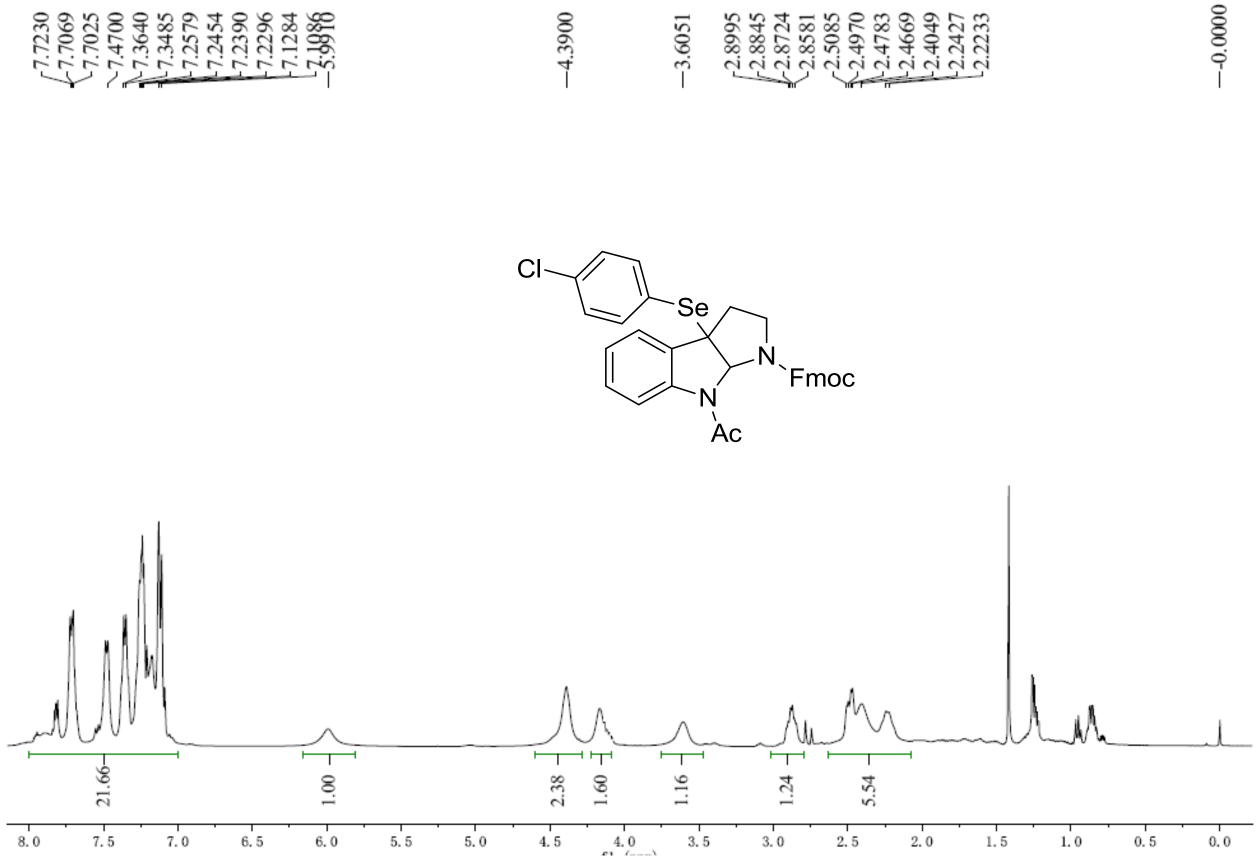
7.7230
7.7069
7.7025
7.4700
7.3640
7.3485
7.2579
7.2454
7.2390
7.2296
7.1284
7.1086
5.9910
4.3900
3.6051
2.8995
2.8845
2.8724
2.8581
2.5085
2.4970
2.4783
2.4669
2.4049
2.2427
2.2233
-0.0000
Cl
Se
N
Fmoc
N
Ac
21.66
1.00
2.38
1.60
1.16
1.24
5.54
8.0 7.5 7.0 6.5 6.0 5.5 5.0 4.5 4.0 3.5 3.0 2.5 2.0 1.5 1.0 0.5 0.0
f1 (ppm)

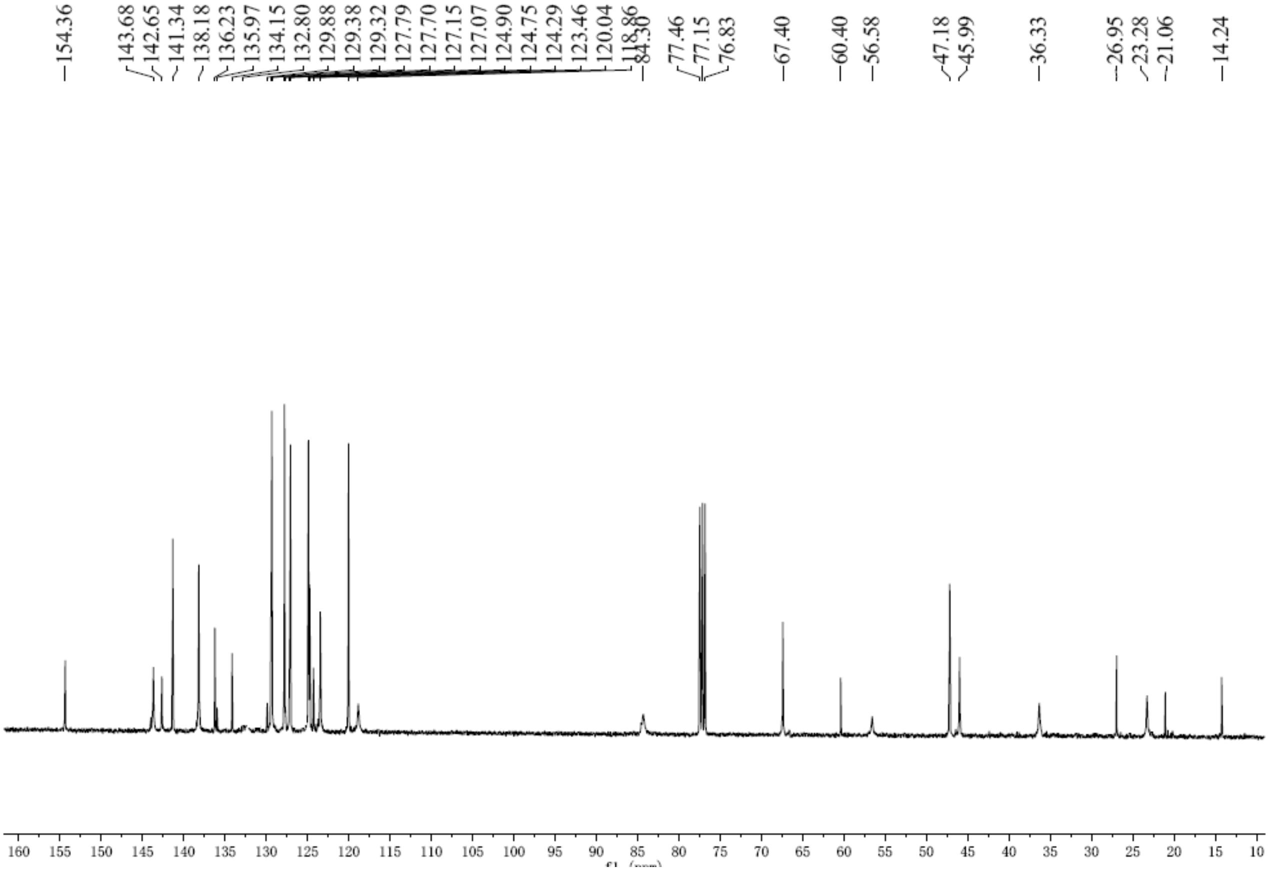
154.36
143.68
142.65
141.34
138.18
136.23
135.97
134.15
132.80
129.88
129.38
129.32
127.79
127.70
127.15
127.07
124.90
124.75
124.29
123.46
120.04
118.86
84.30
77.46
77.15
76.83
67.40
60.40
56.58
47.18
45.99
36.33
26.95
23.28
21.06
14.24
160 155 150 145 140 135 130 125 120 115 110 105 100 95 90 85 80 75 70 65 60 55 50 45 40 35 30 25 20 15 10
f1 (ppm)

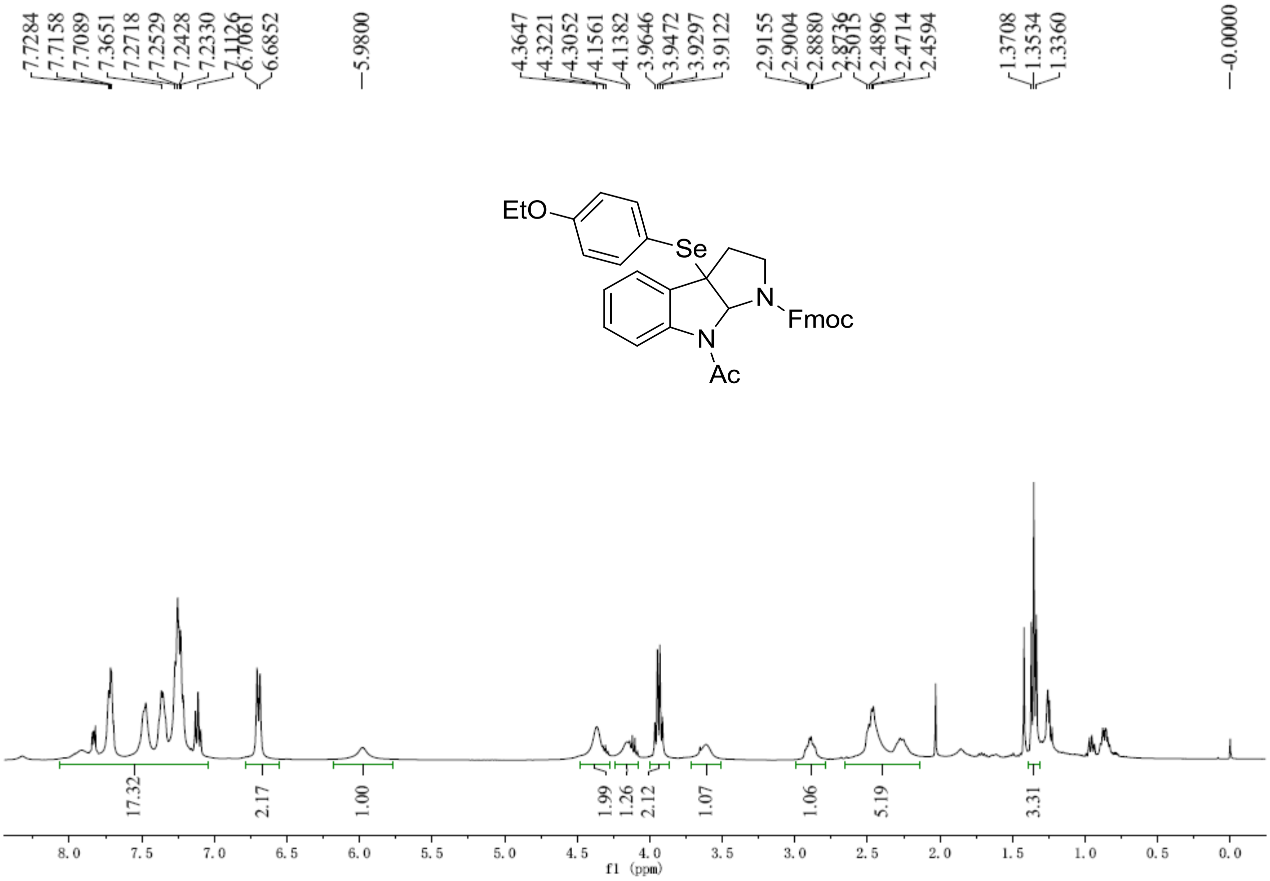
7.7284
7.7158
7.7089
7.3651
7.2718
7.2529
7.2428
7.2330
7.1126
6.7061
6.6852
5.9800
4.3647
4.3221
4.3052
4.1561
4.1382
3.9646
3.9472
3.9297
3.9122
2.9155
2.9004
2.8880
2.8736
2.5015
2.4896
2.4714
2.4594
1.3708
1.3534
1.3360
-0.0000
EtO
Se
N
Fmoc
N
Ac
17.32
2.17
1.00
1.99
1.26
2.12
1.07
1.06
5.19
3.31
f1 (ppm)

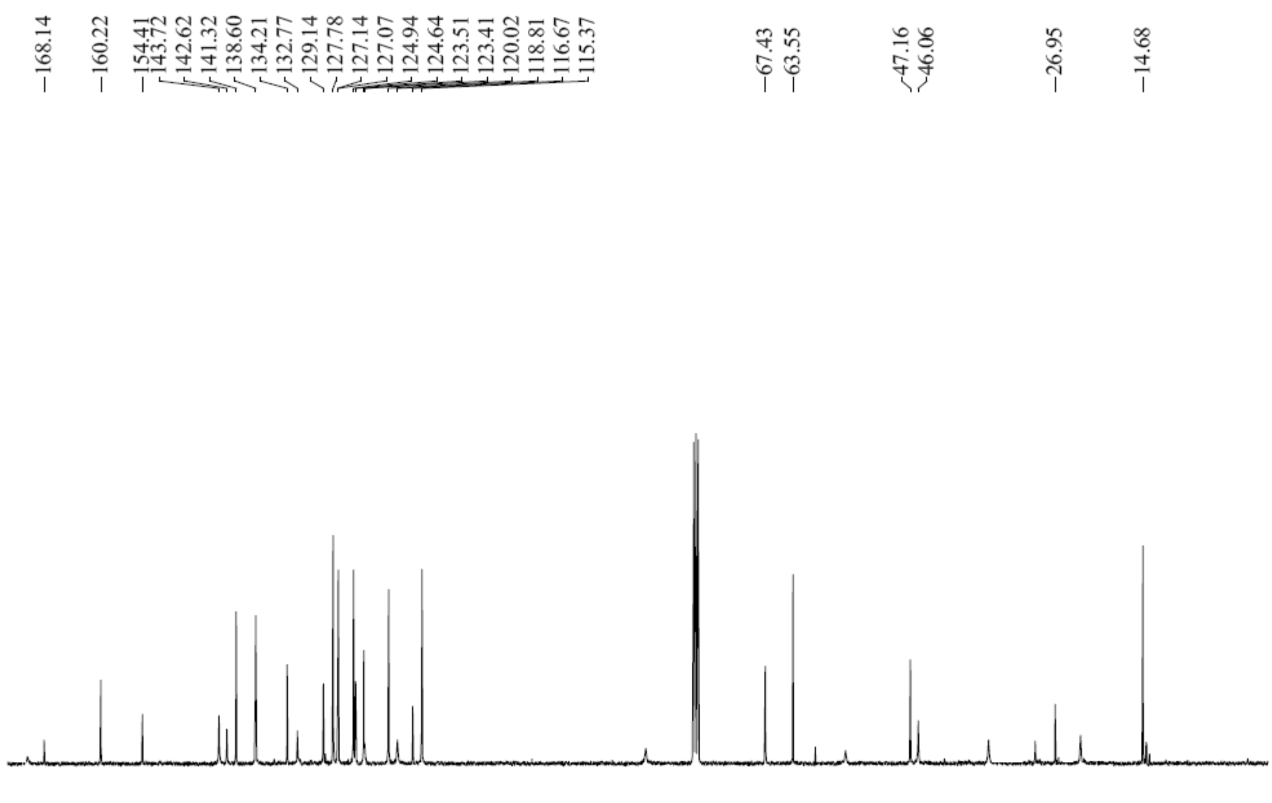
168.14
160.22
154.41
143.72
142.62
141.32
138.60
134.21
132.77
129.14
127.78
127.14
127.07
124.94
124.64
123.51
123.41
120.02
118.81
116.67
115.37
67.43
63.55
47.16
46.06
26.95
14.68
f1 (ppm)

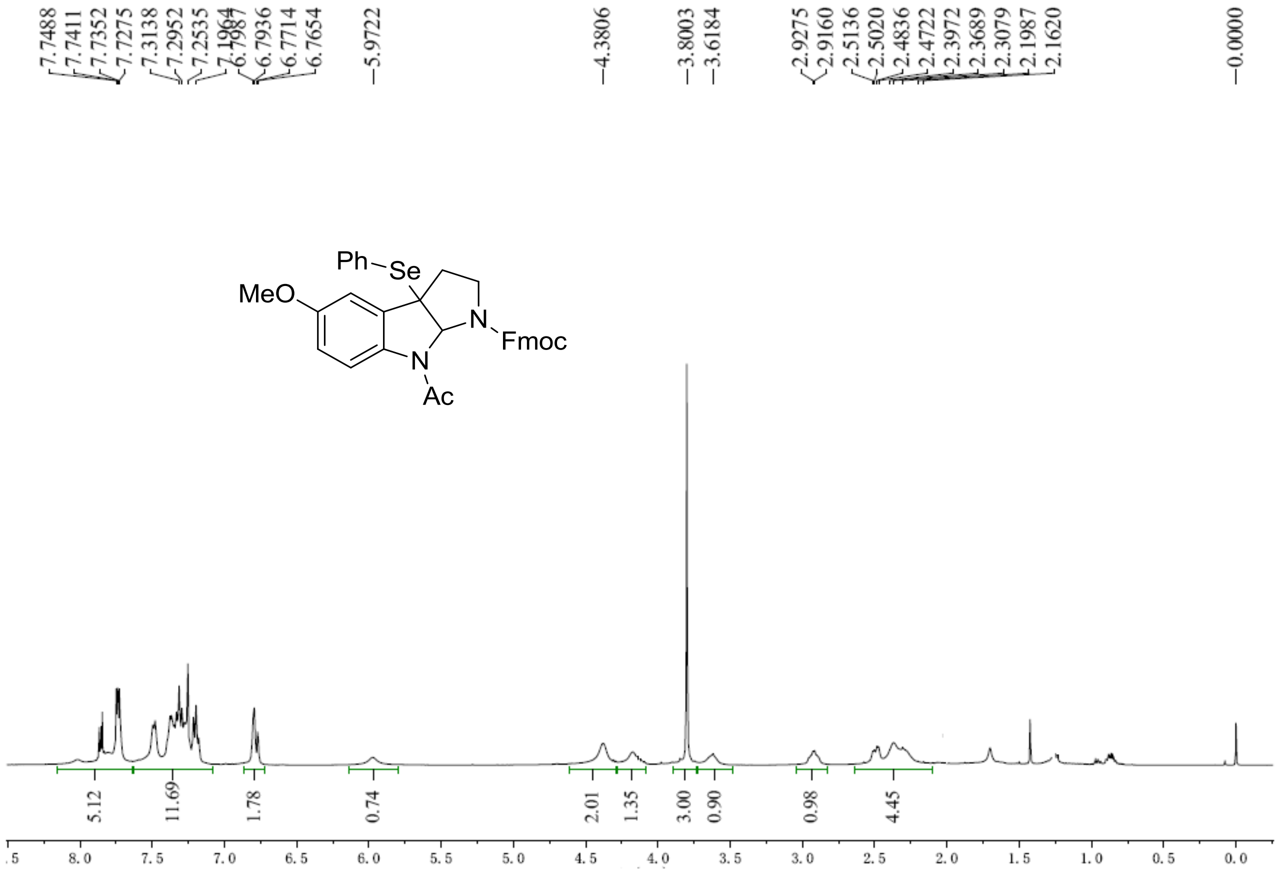
7.7488
7.7411
7.7352
7.7275
7.3138
7.2952
7.2535
6.7936
6.7714
6.7654
5.9722
4.3806
3.8003
3.6184
2.9275
2.9160
2.5136
2.5020
2.4836
2.4722
2.3972
2.3689
2.3079
2.1987
2.1620
0.0000
Ph
Se
MeO
N
Fmoc
N
Ac
5.12
11.69
1.78
0.74
2.01
1.35
3.00
0.90
0.98
4.45

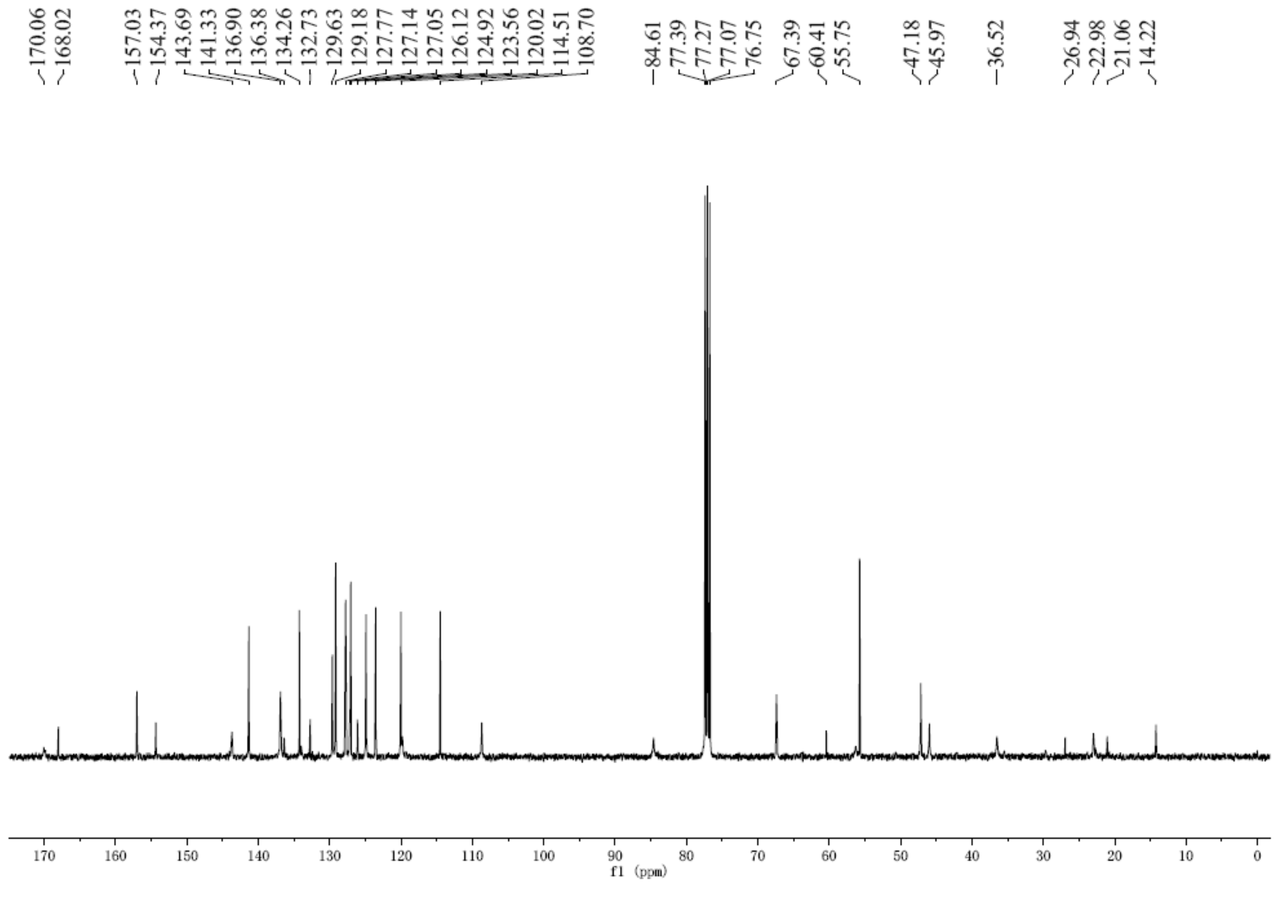
170.06
168.02
157.03
154.37
143.69
141.33
136.90
136.38
134.26
132.73
129.63
129.18
127.77
127.14
127.05
126.12
124.92
123.56
120.02
114.51
108.70
84.61
77.39
77.27
77.07
76.75
67.39
60.41
55.75
47.18
45.97
36.52
26.94
22.98
21.06
14.22
f1 (ppm)

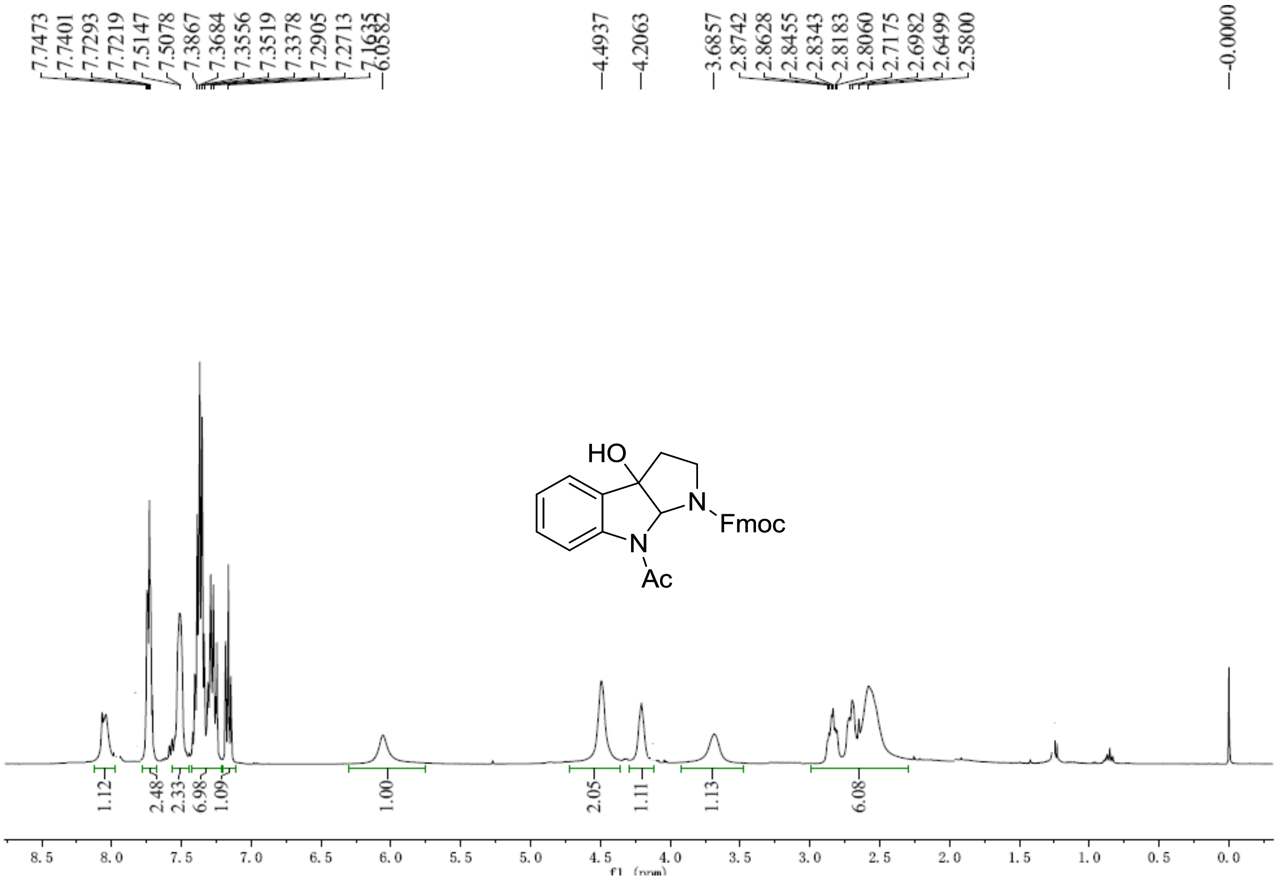
7.7473
7.7401
7.7293
7.7219
7.5147
7.5078
7.3867
7.3684
7.3556
7.3519
7.3378
7.2905
7.2713
7.1635
6.0582
4.4937
4.2063
3.6857
2.8742
2.8628
2.8455
2.8343
2.8183
2.8060
2.7175
2.6982
2.6499
2.5800
-0.0000
HO
N
Fmoc
N
Ac
1.12
2.48
2.33
6.98
1.09
1.00
2.05
1.11
1.13
6.08
f1 (ppm)

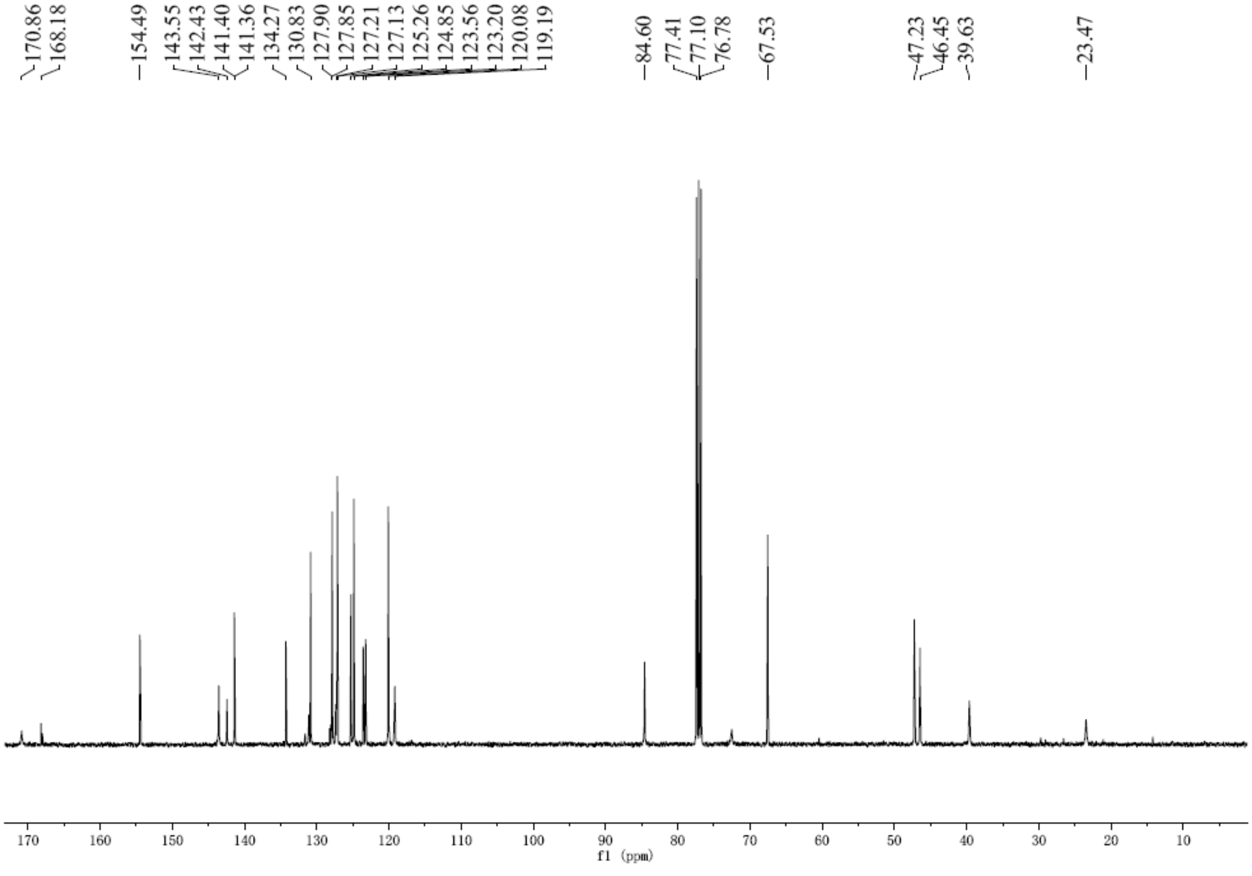
170.86
168.18
154.49
143.55
142.43
141.40
141.36
134.27
130.83
127.90
127.85
127.21
127.13
125.26
124.85
123.56
123.20
120.08
119.19
84.60
77.41
77.10
76.78
67.53
47.23
46.45
39.63
23.47
f1 (ppm)
